# Boosting Electronic Properties of CsPbBr_3_ Nanocrystals via Lithium‐Ion Doping and Surface Passivation for Enhanced Electrical Conductivity and Efficient White Light‐Emitting Diodes

**DOI:** 10.1002/advs.202417304

**Published:** 2025-06-05

**Authors:** Zhongsheng Ge, Siyuan Wan, Muhammad Moin, Sk Abdul Moyez, Lizhuang Dong, Hamood ur Rehman Haris, Marek Piotrowski, Zhiming Wang, Tim Leydecker, Udayabhaskararao Thumu

**Affiliations:** ^1^ Institute of Fundamental and Frontier Sciences University of Electronic Science and Technology of China Chengdu 610054 China

**Keywords:** Conductivity of perovskites, Density functional theory, Li^+^‐doped CsPbBr_3_ NCs, Li_m_Pb_n_‐ligated complexes, White light emitting diodes

## Abstract

Lithium's interaction with CsPbBr_3_ nanocrystals (NCs), can enhancing its intrinsic electrical conductivity (σ) for high‐performance device applications. Herein, two distinctly different modes of Li⁺ interaction with CsPbBr_3_ NCs: minor lattice insertion (0.07% relative to Cs) and predominant surface passivation is reported through Li_m_Pb_n_ alloy formation. In contrast, Li⁺ exhibits significantly reduced interaction with Cs_4_PbBr_6_ NCs, which could be due to the persence of lower amount of Pb^2+^ on the surface of these structures. The σ of CsPbBr_3_:*x*Li^+^ NCs through bottom‐contact devices exhibited a gradual increase from 2.1 × 10^−7^ to as high as 2.5 × 10^−6^ S m^−1^, which is a 50‐fold improvement compared to CsPbBr_3_ NCs. The enhanced σ is attributed to the presence of Li^+^ doping and surface passivation of CsPbBr_3_ by the Li_m_Pb_n_ ligated complexes. DFT calculations revealed electron movement from the valence and to conduction band and a reduced bandgap further supporting the inferences from experimental studies. The unique feature of the increased luminescence and σ of CsPbBr_3_:Li^+^ NCs is explored for fabricating white light emitting diodes. The luminescence efficacy of the device is in the range of 88.5 to 112.5 lm W^−1^ which is higher compared to pure CsPbBr_3_ NCs (96.5 lm W^−1^), offering a pathway for advanced optoelectronic applications.

## Graphical abstract

Lithium doping in CsPbBr_3_ nanocrystals (NCs) enhances electrical conductivity (50‐fold increase) and photoluminescence quantum yield (50% to 67%) via lattice insertion and Li_m_Pb_n_ alloy passivation. Optimized LiBr:PbBr_2_ ratio enable controlled doping, while hydrolyzed LiBr induces a phase transition to Cs_4_PbBr_6_. White LEDs using CsPbBr_3_:Li^+^ NCs showed superior luminous efficiency (up to 112.5 lm W^−1^), advancing optoelectronic applications.

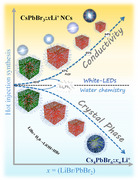

## 1 Introduction

Tuning the electronic properties of CsPbBr_3_ NCs, especially to enhance their electrical conductivity (σ) while maintaining their excellent photoluminescence quantum yield (PLQY) holds promising optoelectronic applications such as light‐emitting diode (LED) and field‐effect transistor (FET) based devices.^[^ ^1^, ^2^, ^3^, ^4^, ^5^, ^6^ ^]^ To achieve this goal, strategies like, optimizing the amount of cation dopant,^[^ ^7^, ^8^, ^9^ ^]^ creation of heterostructures,^[^ ^10^, ^11^, ^12^ ^]^ and surface engineering, have gained substantial attention due to their potential to tune the bandgap, improve charge carrier mobility, life time of the charge carriers and induce magnetic properties.^[^ ^13^, ^14^, ^15^, ^16^ ^]^ Common dopants to replace Pb^2+^ include multivalent ions like Sb^3+^, various lanthanide ions (Ln^3+^, Yb^3+^, Er^3+^), Zn^2+^, Mn^2+^, Fe^2+^, Ca^2+^, Cu^2+^, Cd^2+^, Sn^2+^ and Sr^2+^ have enhanced thermodynamic stability and optical properties of perovskite NCs.^[^ ^17^, ^18^, ^19^, ^20^, ^21^, ^22^, ^23^, ^24^ ^]^ Additionally, substitution of Cs^+^ with ions like Rb⁺, Na⁺, and K⁺ has substantially improved the performance of the doped systems toward white and green LEDs.^[^ ^25^, ^26^, ^27^, ^28^, ^29^, ^30^, ^31^, ^32^, ^33^ ^]^ Two primary factors, namely, the lattice doping and surface passivation contribute to the enhanced performance and stability of NCs. Doping at optimal concentrations has shown enhancement in the PL intensity and moreover surface passivation could also enhance the PL intensity.^[^ ^34^ ^]^

In fact, theoretically, the small metal ions such as Li^+^, Na^+^, and K^+^ as an impurity atom within the lattice destabilizes the perovskite structure.^[^ ^25^, ^35^ ^]^ However, the metal ions such as Rb^+^, K^+^ and Na^+^ follows the process of surface passivation of CsPbBr_3_ NCs for improving photostability, PLQY, and color purity.^[^ ^36^ ^]^ For example, the addition of K‐oleate led to the formation of KBr on the CsPbI_3‐x_Br_x_ NC surface, which effectively passivated the NC surface, achieving a PLQY of over 90%.^[^ ^37^ ^]^ In this context, the interaction of Li⁺ ions (76 pm) with CsPbBr_3_ NCs is of particular interest due to their reversible interactions with electronic materials and their use in energy storage.^[^ ^38^ ^]^ While perovskite oxides have been used as host materials for Li^+^ ions, the smaller lattice plane gaps in CsPbBr_3_ perovskites have limited their use for this purpose. However, the advantage of Li^+^ ions to form alloys with Pb, which could allow clustering of Li^+^ with Pb^2+^ metal ions either on the surface or inside the lattice, potentially alter the intrinsic properties of CsPbBr_3_ NCs and enhance their applicability in LED devices. In case of Na⁺/K⁺ doping, which primarily targets Cs⁺ substitution or surface Br vacancy passivation for PLQY enhancement (>90%),^[^ ^29^, ^39^, ^40^, ^41^, ^42^, ^43^, ^44^, ^45^ ^]^ Li⁺’s smaller radius could enables interstitial lattice doping and strain‐induced quantum confinement. Despite their importance in doping CsPbBr_3_ NCs, studies specifically investigating Li or K doping/passivation in halide perovskites remain scarce, representing only ∼0.9% of total halide perovskite research (from 31,244 results for halide perovskites to just 284 for Li/K‐doped systems). This shows that the field of either Li or potassium passivated halide perovskites is less explored and needs intense research. In particular, lithium doped halide perovskites are appealing due to the changes in their magnetic behavior, Burstein‐Moss bandgap shift, increased photocurrent, battery applications.^[^ ^46^, ^47^ ^]^ It is surmised that the unusual surface passivation modes available by the atomic level interactions of Li and Pb may lead to high radiative efficiency, excellent charge transport and photostable bandgaps and so this study.

Herein, we studied the interaction of Li⁺ with CsPbBr_3_ NCs using the hot injection method to fabricate various CsPbBr_3_:Li NC compositions by progressively increasing the amount of LiBr during the synthesis. The results reveal the possibility of insertion of Li^+^ into lattice (to a minor extent) and the surface passivation (major extent) of CsPbBr_3_ NCs leading to improved optical and electronic properties. The interaction of Li^+^ with Cs_4_PbBr_6_ NCs and CsPbBr_3_ NCs is compared. Such an intereaction was found to be lower in the case of the former compared to the latter which is attirbuted to the surface rich in Cs^+^ species in the former case. The intrinsic σ of these CsPbBr_3_:Li^+^ NCs was measured. A 50‐fold increase in the σ of CsPbBr_3_:Li^+^ NCs compared to that of the pure CsPbBr_3_ NCs was observed. Additionally, the PLQY values of CsPbBr_3_:Li NCs were found to be higher (50% to 67%) than those of pure CsPbBr_3_ NCs. The role of Li^+^ doping, the formation of Li_m_Pb_n_ alloy species, the improvement in the electrical conductivity, and stability of the doped NCs on the enhancement of the performance of CsPbBr_3_:Li NC in white LEDs (luminiscence efficacy and PLQY) was studied in detail and new insight was provided.

## 2 Results and Discussion

**Figure 1:**
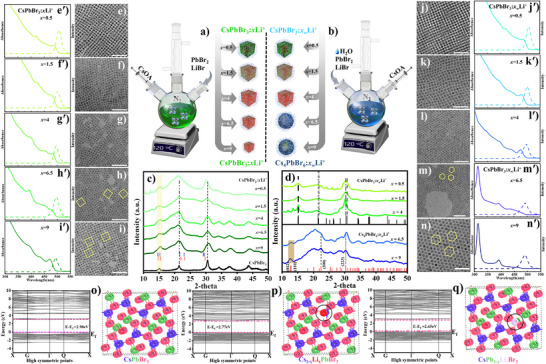
Schematic representation of the synthetic process of CsPbBr_3_:*x*Li^+^ NCs and Cs_4_PbBr_6_:*x_w_* Li^+^ NCs through hot injection procedures under dry (a) and wet conditions (b). The ratio between LiBr:PbBr_2_ is varied as labelled (*x* = 0.5 to 9). The size and phase changes in NCs are schematically represented (top to bottom). Red colored dots within green colored nanocubes indicates the Li^+^ doping in the NC's lattice, and the complete red colored dots indicates the surface passivated NCs; blue colored spherical shapes represent the Cs_4_PbBr_6_:*x_w_* Li^+^ (b); XRD pattern of pure CsPbBr_3_ NCs, and CsPbBr_3_:*x*Li^+^ (*x* = 0.5 to 9) NCs prepared at dry conditions (c); XRD pattern of CsPbBr_3_:*x_w_* Li^+^, (*x* = 0.5, 1.5 and 4) NCs and Cs_4_PbBr_6_:*x_w_* Li^+^ NCs, (*x* = 6.5 and 9) prepared at wet conditions (d); (blue line is Li_22_Pb_5_#01‐071‐9500, red line is Li_17_Pb_4_#00‐031‐0687 in the Figure 1c). TEM images (e‐i) and (j‐n) represent the NCs prepared under dry (left panel) and wet conditions (right panel). and their absorption spectra and PL emission spectra are also presented (eʹ‐iʹ) and (jʹ‐nʹ). The scale bar in all the images corresponds to 50 nm. Optimized crystal structure of pristine system, band‐structure of supercell of (o) for CsPbBr_3_, (p) Cs_1‐_ *_x_* Li*_x_* PbBr_3_ and (q) CsPb_1‐_ *_x_* Li*_x_* Br_3_ are presented.

The previously reported hot‐injection synthesis method was used to fabricate the perovskite NCs and to systematically investigate the doping of Li^+^ ions in the lattice of CsPbBr_3_ and surface passivation properties of Li_m_Pb_n_ alloy species.^[^ ^48^ ^]^ Detailed synthetic conditions are provided in S1 (Tables S1 and S2, Supporting Information). In a typical synthesis, under anhydrous conditions, LiBr and PbBr_2_ salts were dissolved in the presence of oleic acid (OA) and oleylamine (OLAM) in octadecene (ODE) at ≈ 100 °C under vacuum for 1 h to remove moisture, after which pre‐heated Cs‐oleate was promptly injected into the reaction mixture at 120 °C under nitrogen atmosphere, leading to the formation of CsPbBr_3_:Li^+^ NCs. In this synthesis, we varied the amounts of LiBr and PbBr_2_ to achieve different molar ratios of LiBr/PbBr_2_ ranging from 0 to 0.5. 1.5, 4, 6.5, 9. For simplicity, the resulting materials were denoted as CsPbBr_3_:*x*Li^+^ NCs (where *x* = 0.5, 1.5, 4, 6.5, and 9 times the LiBr to PbBr_2_ during the synthesis). Since LiBr served as the source of Li^+^ ions, the Br content also increased during the synthesis. Concurrently, the Pb/Br molar ratio too was varied from 1:3 to 1:20 (**Figure** **1**a). To elucidate the role of moisture on the structure and properties of the resulting perovskite materials, LiBr was initially exposed to 5 µL of water. The hydrolyzed LiBr and PbBr_2_ were dissolved with the ligands (ODE, OA, OLAM), and vacuum‐dried at 100 °C to remove the water content, and then Cs‐oleate was injected following the regular hot injection method (Figure 1b). This approach allowed to study the influence of water on the surface engineering and phase transformation processes, providing insights into the final composition of the Li^+^ doped perovskites. The NCs obtained under these conditions are labeled as CsPbBr_3_:*x_w_* Li^+^ NCs, where *x_w_* represents the molar ratio of ${\left(\mathrm{LiBr}/\mathrm{PbB}r_{2}\right)}_{H_{2}O}$ (Figure 1b).

The phase and structural changes in NCs:*x*Li^+^ and NCs:*x_w_* Li^+^ were further analyzed through X‐ray diffraction (XRD) analysis. For samples prepared under dry synthesis conditions the XRD patterns, corresponding to CsPbBr_3_:*x*Li^+^, remained constant for all the molar ratios of LiBr/PbBr_2_ (Figure 1c). The observed diffraction peaks aligned well with the reference orthorhombic CsPbBr_3_ phase. The major difference between XRD patterns of the pure CsPbBr_3_ (Figure 1c) and the Li modified systems, CsPbBr_3_:*x*Li^+^ NCs lies in the peak widths. The peaks at the 2θ vaulues of 15° and 21° are notably broader in the Li‐doped samples compared to the pure CsPbBr₃ nanocrystals. This peak broadening suggests the formation of Li‐Pb clustering within the CsPbBr₃ lattice, likely due to either lattice insertion or surface passivation. The theoretical pattern of Li_22_Pb_5_ (blue line) and Li_17_Pb_4_ (red line) are presented in Figure 1c. These peaks match well with that of CsPbBr_3_:*x*Li^+^ NC's peaks located at 2θ = 15° and 21° resulting in an increase in the peak width. The potential of the synthetic methodology for inserting Li^+^ into the lattice of CsPbBr_3_ was further examined using ^7^Li static NMR spectroscopy of CsPbBr_3_:*x*Li^+^ (x = 0.5) which shows a prominent peak at 0.25 ppm, corresponding to presence of Li^+^ ions in CsPbBr_3_ NCs (Figure S1a, Supporting Information). In addition, NMR studies (^1^H, ^13^C, and COSY) are performed on these NCs (Figures S1b and S2, Supporting Information), and the results are matching the previous literature of CsPbBr_3_ NCs.^[^ ^49^ ^]^ Where OLAM bonded strongly and nearly intact nature of OA resonances indicate that presence of free OA in the ligand shell of CsPbBr_3_:*x*Li^+^ (x = 0.5). The LiPb‐OA/OLAM complexes formed during synthesis not only stabilize the nanocrystal surface but also enhance solubility, preventing aggregation. In contrast, the synthesis under wet conditions revealed notable changes as the LiBr/PbBr_2_ molar ratio was varied. A gradual phase transformation from CsPbBr_3_:*x_w_* Li^+^ (*x* = 0.5) to Cs_4_PbBr_6_:*x_w_* Li^+^ (*x* = 9) was observed. For the first three compositions, CsPbBr_3_:*x_w_* Li^+^ (*x* = 0.5, 1.5, 4), the NCs maintained their orthorhombic structure. With increasing LiBr/PbBr_2_ molar ratios, a gradual rise in the intensity of the diffraction peaks at the 2θ = 12° was observed and the relative intensity of the peaks corresponding to CsPbBr_3_ phase decreased. This suggests that the formation of the Cs_4_PbBr_6_:*x_w_* Li^+^ (*x* = 6.5 and 9) phase became more prominent at higher Li^+^ concentrations, suppressing the crystallization of the primary CsPbBr_3_ phase (Figure 1d).

The structural changes were further confirmed by transmission electron microscopy (TEM) analysis, as presented in Figure 1 (e–n). The TEM images of CsPbBr_3_:*x*Li^+^ (*x* = 0.5–9) showed that the NCs synthesized under dry conditions consistently exhibited a cubic or psedo cubic structure with various LiBr/PbBr_2_ molar ratios. This structural stability suggests that the presence of Li^+^ ions during the synthesis did not significantly disrupt the overall perovskite structure (Figure 1 e–i). The particle sizes varied from 7 nm to 6 nm for *x* = 0.5 to 9 (Figure S3, Supporting Information). This indicates that the particle sizes are nearly similar but with minor changes in their morphologies. At lower LiBr concentrations the NCs are cubic in nature (Figure 1e–g), whereas at higher concentrations, the shape is nearly cubic along with pseudo‐spherical morphologies, as seen in the TEM (Figure 1h–i and STEM image of Figure S4, Supporting Information). On the other hand, the samples prepared under wet conditions displayed a gradual transition from cubic nanostructures (*x_w_* = 0.5 to 4) to spherical NCs (*x_w_* = 6.5 to 9). The elemental compositions of CsPbBr_3_:9Li NCs are Cs:Pb:Br = 1:0.9:2.4, and for Cs_4_PbBr_6_ are about 4:1:7.2, according to elemental compositions (Figure S5, Supporting Information). This indicates a phase transformation from CsPbBr_3_ to Cs_4_PbBr_6_ phase, as corroborated by the XRD results. Interestingly, the self‐assembly process was initiated when the Cs_4_PbBr_6_:9*_w_* Li^+^ NCs possessed uniform diameter with 8.09 ± 0.47 nm and when the solvent was evaporated on the TEM grid. The NC solution was slowly driven to supersaturation by increasing the concentration upon hexane evaporation on the TEM grid, where the sample possessed highly ordered 2D and 3D Cs_4_PbBr_6_:9*_w_* Li^+^ NCs superlattices composed of uniform single NCs with hexagonal close packed (hcp) superlattices (Figures S6 and S7, Supporting Information) with an interparticle separation of 2.2 nm, related to OA and OLAM. The generation of such unique superlattices of Cs_4_PbBr_6_ NCs is attributed to the highly monodisperse NCs with preferred ligand structure obtained in this synthetic conditions.^[^ ^50^, ^51^ ^]^

The impact of the interaction of Li^+^ ions with the CsPbBr_3_ NCs on the optical properties is examined. Under dry reaction conditions (Figure 1eʹ–iʹ), the absorption spectra showed a blue shift compared to the pure CsPbBr_3_ NCs synthesized under similar conditions (hot injection at 120 °C). The absorption and emission maxima for pure CsPbBr_3_ NCs present at 510 nm and 524 nm as shown in Figure S8 (Supporting Information). At lowest feeding level of Li^+^, CsPbBr_3_:*x*Li^+^ NCs (*x* = 0.5) exhibited absorption and emission maxima at 490 nm and 505 nm. The absorption maxima gradually blue shifted with increased feeding of Li^+^ into the system, from 495 nm to 483, 478, 475, and 470 nm, respectively, as *x* changed from 0.5 to 9. The PL spectra of the NCs synthesized under dry conditions showed a slight blue shift with the increase in the LiBr. This observation is consistent with the changes observed in the absorption spectra as a result of quantum confinement arising from reduction in NC size from 8 nm to 6 nm. However, we noticed a slight hump at 400 nm, corresponding to smaller NCs (below 6 nm), as observed in the particle size distribution deduced from the TEM images (Figure S3a–e, Supporting Information). These were not considered in the analysis as they did not contribute to the PL emission, indicating a low population. At the same time, the PL emission peaks ranged from 508 nm to 490 nm, with the linewidth narrowing from 25 to 21 nm (Table S3, Supporting Information), indicating improved size and compositional homogeneity of the NCs.

In contrast, the NCs synthesized under wet conditions (Figure 1jˊ–lˊ) exhibited different absorption properties with increase in *x_w_*. When *x* = 0.5 to 4 maintained a similar absorption to that of the NCs synthesized under dry conditions. However at higher LiBr amounts, where *x* = 6.5 to 9, the samples showed the emergence of an additional absorption peak at approximately 320 nm. This peak is attributed to the formation of the Cs_4_PbBr_6_:*x_w_* Li^+^ NC phase, matching well with the earlier XRD and TEM observations. This phase change leads to a significant blue shift in the absorption and PL emission properties, as the Cs_4_PbBr_6_:*x_w_* Li^+^ NCs phase has a wider bandgap compared to the parent CsPbBr_3_ phase.

**Figure 2:**
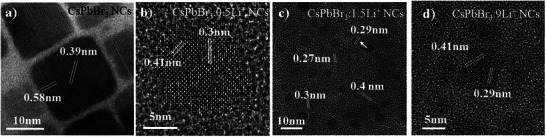
a–d) shows the HRTEM images of pure CsPbBr_3_, CsPbBr_3_:0.5Li^+^, CsPbBr_3_:1.5Li^+^, and CsPbBr_3_:9Li^+^, respectively.

To understand these optical properties and related electronic band structures, the hybrid generalized gradient approximation (GGA) using the Perdew‐Burke‐Ernzerhof (PBE) functional simulation study was employed for 6% (one Li atom per 16 Pb atoms in a supercell) of Li^+^ ions substituted for Cs^+^ and Pb^2+^ ions in orthorhombic CsPbBr_3_ NCs, with a 2 × 2 × 1 supercell. First, the intrinsic characteristics of the band structures of the pure CsPbBr_3_ compound and the Li^+^ doped systems were explored. Figure 1 (o‐q) illustrates the electronic band structures of both pure and Li‐doped Cs_1‐_ *_x_* Li*_x_* PbBr_3_ and CsPb_1‐_ *_x_* Li*_x_* Br_3_ perovskites, showing their behavior along the high‐symmetry points in the first Brillouin Zone (FBZ). The results confirm that these materials exhibit direct bandgaps, with the conduction band minimum (CBM) and valence band maximum (VBM) both located at the Γ point for the pure and Li‐doped structures. The bandgap calculations indicate that the incorporation of 6% of Li leads to significant alterations, as shown in Figure 1 (o‐q). Doping with Li^+^ decreases the energy gap from 2.98 (for CsPbBr_3_) to 2.77 eV, while substituting Pb atoms with Li reduces the gap further, from 2.98 to 2.65 eV. The introduction of Li into CsPbBr_3_ can create impurity energy levels within the bandgap, acting as intermediate states that lower the energy required for electron transitions between the valence and conduction bands. These results are in good agreement with the experimentally observed optical properties. As a Group I element, Li donates electrons to the host material, increasing the electron density in the conduction band and further lowering the bandgap. Additionally, Li incorporation can cause lattice distortions, influencing the material's electronic properties. For instance, Rb^+^‐doped CsPbBr_3_ or mixed‐halide Cs_1–x_PbBrI_2_ NCs with different ratios of Rb/Cs showed a gradual increase in the bandgap and thus the photoluminescence blue shifts from green to blue with the increase of the Rb/Cs ratio.^[^ ^27^, ^52^, ^53^ ^]^ The authors attributed the increase in the bandgap to changes in the valence and conduction bands caused by the decrease of in‐plane Pb‐Br‐Pb bond angle of the [PbBr_6_] octahedron by the replacement of Cs^+^ with smaller Rb^+^ ions that does not fit well into perovskite lattice due to low tolerance factor. From the HRTEM of the pure phase CsPbBr_3_ NCs, we observed lattice spacings of 0.39 nm and 0.58 nm, corresponding to the (210) and (101) crystal plane spacings, respectively (**Figure** **2**a–d). The lattice spacings were found to reduce ≈0.3 nm (210) and 0.41 nm (101). This lattice contraction is due to smaller Li^+^ (76 pm) possibly replace with Pb^2+^ (119 pm), and is consistent with previous reports where lattice contraction occurs upon metal ion doping in CsPbBr_3_ and these results were in consistent with the ^7^Li NMR.^[^ ^54^, ^55^, ^56^, ^57^, ^58^ ^]^ Furthermore, we observed the formation of nanoscale clusters within the NCs, exhibiting a characteristic lattice spacing of 0.29 nm (indicated by arrows in Figure 2c).

ICP‐MS analysis (Table S6, Supporting Information) confirms the presence of Li in doped samples, though quantitative correlation with Pb remains challenging due to Li's low atomic mass. In correlated to the XRD data (Figure 1c) broadening of the (110) peak and a slight (≈1%) expansion in the (200) reflection spacing, suggesting lattice distortion induced by Li incorporation. The persistence of tetragonal/orthorhombic splitting alongside general peak broadening with increasing Li content further supports structural perturbation. Notably, the broader peaks in Li‐doped NCs compared to undoped CsPbBr_3_ may indicate either heterostructure formation or surface passivation by LiPb‐ ligated (OA/OLAM) complexes (Figure S10, Supporting Information), analogous to reported cases of Ag, Na, or K‐oleate passivation in perovskites (Figure S9, Supporting Information).^[^ ^59^ ^]^ Charge balance considerations (2Li^+^ ≈ 1Pb^2+^) and ionic radii (Cs^+^ > Pb^2+^ > Li^+^) further support the likelihood of Li‐Pb clustering over direct Pb^2+^ substitution.

To further probe the formation of Li_m_Pb_n_ species, XRD and HRTEM was performed on three samples: a pre‐characterized Li_17_Pb_4_ alloy material, LiPb‐ligated complexes and CsPbBr_3_:9Li⁺ NCs. First, a XRD measurements are performed on Li‐, Pb‐, and mixed metal LiPb‐ligated complexes (S1, Supporting Information). The spectral analysis revealed characteristic overlap between the LiPb‐complexes and the constituent Li‐ and Pb‐complexes, verifying the incorporation of both metal species in the LiPb‐complexes (Figure S10, Supporting Information). HRTEM characterization of the LiPb‐complexes revealed lattice spacings measuring ≈0.29 nm (Figure S11, Supporting Information). HRTEM analysis of CsPbBr_3_:9Li NCs appeared as heterostructures reveals a characteristic 0.29 nm lattice spacing (Figure S12, Supporting Information), which shows striking consistency with two reference systems: 1) the LiPb‐complexes, and 2) pre‐characterized Li_17_Pb_4_ alloy clusters (Figure S13, Supporting Information). This three‐way correspondence in lattice parameters strongly suggests that similar Li‐Pb alloy formation occurs in all cases, with the 0.29 nm spacing serving as a distinctive fingerprint for these mixed metallic phases within the perovskite matrix. All samples exhibited a prominent lattice spacing of 0.29 nm (Figure S13, Supporting Information), consistent with the (226) plane of Li_17_Pb_4_ (d_theory_ = 0.29 nm, ICSD 107216), though its diffraction intensity is notably low. This suggests either preferential orientation damping high‐order reflections or local structural deviations (e.g., Li^+^ disorder). Thus, based on the revelations from the XRD (Figures S9 and S10, Supporting Information), HRTEM (Figure 2; Figures S11–S13, Supporting Information) and ICP‐MS analysis it is surmised that Li_m_Pb_n_ type species are formed in Li‐doped perovskites, potentially tuning optoelectronic properties via strain or band alignment.

The PL lifetime data on CsPbBr_3_:*x*Li^+^ NCs (*x* = 4 and 9), CsPbBr_3_:4*_w_* Li^+^ NCs, and Cs_4_PbBr_6_:9*_w_* Li^+^ NCs samples under dry and wet conditions are compared (Figure S14, a,b, Supporting Information compared) along with pure CsPbBr_3_ NCs.^[^ ^60^ ^]^ The average lifetime (τ_avg_) of NCs increased from CsPbBr_3_:4*_w_* Li^+^ NCs to Cs_4_PbBr_6_:9*_w_* Li^+^ NCs films prolonged from 3.89 to 9.61 ns. Increased lifetime values were also observed in previously reported dual‐phase CsPbBr_3_/Cs_4_PbBr_6_ hybrid NCs, which can be linked to the shallow trap states near the edge of the bandgap.^[^ ^61^ ^]^ This implies the possibility that Cs_4_PbBr_6_:9*_w_* Li^+^ NCs are composed of hybrid nature along with the CsPbBr_3_ inclusions. Additionally, the lower lifetime (τ_avg_ ≈3 ns) compared to pure CsPbBr_3_ NCs (τ_avg_ ≈18 ns) usually indicates the suppression of nonradiative decay, and the generated excitons are more prone to recombine via radiative pathways.

**Figure 3:**
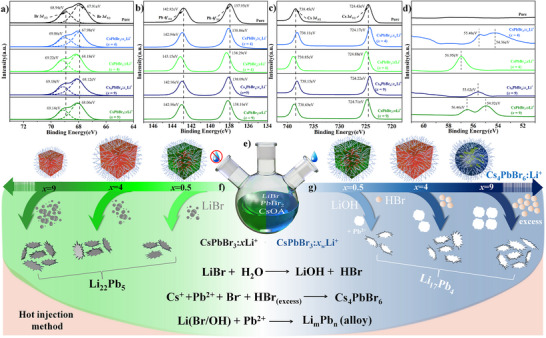
X‐ray photoelectron spectra of the CsPbBr_3_:*x*Li^+^ NCs (*x* = 4 and 9) and the NCs prepared at wet conditions CsPbBr_3_:*x_w_* Li^+^ NCs (*x_w_* = 4) and CsPbBr_3_:*x_w_* Li^+^ NCs (*x* = 9). High resolution XPS spectra of the Br 3*d* (a), Pb 4*f* (b), Cs 3*d* (c), and Li 1*s* (d). The schematic illustration of the synthesis process under dry and humid conditions, and the formation of by‐product Li_m_Pb_n_ (e‐g). Schematic representation of green colored NCs with red dots indicating the clustering of Li_m_Pb_n_ within the lattice. And red colored NCs indicate the surface passivation of NCs by Li‐oleate or CsPbBr_3_‐Li_m_Pb_n_ alloy formation.

X‐ray photoelectron spectroscopy (XPS) analysis of the CsPbBr_3_:*x*Li^+^ NCs provides valuable insights into the elemental composition and the nature of lithium interaction with the perovskite lattice. The presence of Li^+^ ions in these NCs systems was primarily confirmed through XPS, as traditional scanning electron microscopy energy dispersive spectroscopy (SEM‐EDS) struggles to detect Li^+^ due to its low characteristic radiation energy.^[^ ^62^ ^]^ The XPS analysis was conducted on four different samples, including CsPbBr_3_:*x*Li^+^ NCs (*x* = 4 and 9), CsPbBr_3_:*x_w_* Li^+^ NCs (*x_w_* = 4), and Cs_4_PbBr_6_:*x_w_* Li^+^ NCs (*x_w_* = 9) (**Figure** **3**).

The XPS spectra were calibrated using the C 1*s* peak at 285.0 eV. In all the four samples, the Li 1*s* peak was observed at the binding energy values of (54.42–56.42 eV), indicative of the presence of Li^+^ ions in the perovskite system (Figure 3d). From the quantification XPS peak areas, the percentage of Li^+^ in CsPbBr_3_:4Li^+^ and CsPbBr_3_:9Li^+^ NCs was found to be 0.15% and 0.36% with respect to Cs. Whereas, in the case of CsPbBr_3_:4*_w_* Li and Cs_4_PbBr_6_: 9*_w_* Li NCs, the presence of Li^+^ was 0.19% and 0.07% respectively (Table S4, Supporting Information). The relatively lower amount of Li content (0.07%) in Cs_4_PbBr_6_: 9*_w_* Li compared to CsPbBr_3_:4*_w_* Li (0.19%) is attributed to the sudden change in the phase of the perovskite material beyond x = 4 in the CsPbBr_3_:x*_w_* Li systems and the chemical composition as well as the structural features and their properties are intimately related as expected. The amount of Li^+^ doping in these NCs is much lower compared to the literature values for other metal ions (e.g., Fe^2+^, Rb^+^, Bi^3+^, Ca^2+^, Cu^2+^, Zn^2+^) (Table S5, Supporting Information).

The binding energy of Li^+^ in CsPbBr_3_:4Li^+^ NCs was found to be ≈ 56.8 eV, which is consistent with the binding energy range of Li^+^ in LiBr (56.0–56.8 eV). This observation supports that the Li^+^ ions interacting with Br by replacing Cs^+^ ions and closely connected with Pb^2+^ ions within the lattice (heterostructure such as CsPbBr_3_‐Li_m_Pb_n_ is proposed in the later section). In CsPbBr_3_:9Li^+^ NCs, the Li 1*s* binding energy decreased to 54.9 eV, indicating the presence of Li^+^ ions not in the lattice, potentially in a carboxylate form associated with oleate ions (binding energy of Li_2_CO_3_ at 54.9 eV).^[^ ^62^ ^]^ In the case of wet conditions, for the CsPbBr_3_:4*_w_* Li^+^ NCs, the Li 1*s* peak was observed at 54.4 eV which is lower than that expected for Li^+^ in LiBr, aligning more closely with binding values for LiOH. This suggests that Li^+^ is present on the surface of CsPbBr_3_ NCs and shows another peak at 55.5 eV indicating the bulk of the lattice contains LiBr. In the case of Cs_4_PbBr_6_:9*_w_* Li^+^ NCs, the Li^+^ binding energy value is around 55.6 eV, nearly matching with that of the LiOH species on the surface of Cs_4_PbBr_6_ NC lattices.

The high‐resolution XPS spectra of Cs 3*d*, Pb 4*f*, and Br 3*d* revealed insights into the interactions between Li^+^ and the surrounding elements, whether it is replacing Cs^+^ ions or Pb^2+^ by analyzing their binding energy values (Figure 3a–c). The structural integrity of CsPbBr_3_ remained largely unaffected by the presence of Li^+^. In CsPbBr_3_:4Li^+^ NCs, the Br 3*d*_5/2_ peak exhibited a slightly higher energy shift (0.3 eV) compared to pure CsPbBr_3_, indicating that the introduction of Li^+^ affects the local electronic environment of bromine. This shift is consistent with the reported binding energy values for CsPbBr_3_ (Br 3*d*_5/2_ at 67.9 eV), suggesting that while Li^+^ carries a higher charge density than Cs^+^, the overall impact on the Br environment is modest. However, the shift is minimal, as it is only 0.15% of Li^+^ doped into the system (based on the XPS quantification). Similarly, the Pb 4*f*_7/2_ peak for the same NCs shows a shift of 0.33 eV compared to pure CsPbBr_3_, which is the largest shift compared to the other three samples, while the Cs 3*d*_5/2_ peak experienced a shift of 0.45 eV. These shifts suggest that Li^+^ may replace either Cs^+^ or Pb^2+^ or interact with Pb to form Li_m_Pb_n_ alloy which could cause a greater shift in the Cs^+^ environment. In the CsPbBr_3_:9Li^+^ NCs sample, the shifts in Br 3*d*_5/2_, Pb 4*f*_7/2_, and Cs 3*d*_5/2_ were minimal (Br 3*d*_5/2_−0.12 eV, Pb 4*f*_7/2_−0.20 eV, Cs 3*d*_5/2_‐0.28 eV) compared to pure CsPbBr_3_, suggesting that the incorporation of Li^+^ at this ratio has a limited effect on the bulk lattice structure, reinforcing the idea that Li^+^ primarily affects the surface composition in the form of Li‐oleate and Li_m_Pb_n_ alloy. In the case of the water‐introduced samples, hydrolysis effects were observed. For the sample, CsPbBr_3_:4*_w_* Li^+^, a decrease in binding energy for Cs^+^ (0.2 eV) and Pb (0.11 eV) was observed, indicating surface hydrolysis. This aligns with the presence of species such as LiOH (from Figure 3d), suggesting that exposure to water alters the surface of the NCs. For Cs_4_PbBr_6_:9*_w_* Li^+^ NCs, the Br 3*d*_5/2_ peak did not show significant shifts, indicating the presence of CsPbBr_3_ inclusions, which is in agreement with the optical properties discussed in the above sections. Chen *et al.* reported that the presence of CsPbBr_3_ inclusions in Cs_4_PbBr_6_ does not show a significant shift in the Br environment.^[^ ^63^ ^]^ Furthermore, the quantitative analysis shows that only 0.06 wt % of Li^+^ was inserted into Cs_4_PbBr_6_ lattice, indicating that the Li^+^ ions interact less with this lattice due to less chance for the formation of surface Li_m_Pb_n_ alloy, as the surface plane is more exposed for Cs^+^ ions. As a result, Cs_4_PbBr_6_ remains unchanged and undoped, highlighting a clear distinction between how Li^+^ behaves in CsPbBr_3_ compared to Cs_4_PbBr_6_. Overall, the XPS analysis of CsPbBr_3_ or Cs_4_PbBr_6_:Li^+^ NCs reveals that only limited Li^+^ ions can partially replace Cs^+^/Pb^2+^ ions without particular selection due to the tendency to form Li_m_Pb_n_ alloy, which could alter the electronic properties and stability of the NCs. Hence, the major mode of interaction of Li^+^ with CsPbBr_3_ is either in the form of surface passivation or clustering with Pb, with doping phenomena being the minor (Figure 3e,g).

As shown in the scheme (Figure 3e, right part), with an increase in the $\;{\left(\mathrm{LiBr}/\mathrm{PbB}r_{2}\right)}_{H_{2}O.}$, the reaction produces HBr as a side product. This HBr content increases with the increase in the quantity of LiBr (equation in Figure 3e,g). In the presence of excessive HBr, the crystallization of Cs_4_PbBr_6_:Li^+^ NCs, rather than CsPbBr_3_ NCs is favored. This indicates that the hygroscopic nature of the LiBr precursor becomes a critical factor during the synthesis leading to the formation of the 0D Cs_4_PbBr_6_:*x_w_* Li^+^ phase, which exhibits blue emission compared to the parent CsPbBr_3_ phase. Furthermore, a white precipitate was obtained as a by‐product from the as synthesized NC solutions, which, upon drying followed by XRD analysis showed the presence of Li_17_Pb_4_ and Li_22_Pb_5_ with a small amount of cesium oleate (Figure S9, Supporting Information). Li_m_Pb_n_ type compounds with typical composition of Li_17_Pb_4_ and Li_22_Pb_5_ are useful for battery applications.^[^ ^64^, ^65^ ^]^ The formation of the Li_17_Pb_4_ and Li_22_Pb_5_ alloys species as a by‐product provides valuable insights into the complex interactions between the various ions (Cs^+^, Pb^2+^, and Li^+^) and the resulting material properties.

Under strictly anhydrous conditions, the Li^+^ ions can effectively substitute Cs^+^ or forming the Li_m_Pb_n_ alloy species within the lattice, potentially leading to improved electrical conductivity and enhanced optical properties of the CsPbBr_3_ NCs. These findings highlight the delicate balance between doping and clustering behavior, phase composition, and morphological characteristics of the Cs‐Pb‐Br perovskite system, which can be carefully tuned by controlling the LiBr/PbBr_2_ molar ratios and the presence or absence of water during synthesis. Importantly, these CsPbBr_3_:*x*Li^+^ NCs are stable at ambient conditions and show no phase changes, as confirmed by their intact nature of absorption spectra for months. Additionally, the NCs are photostable upon illuminated with solar light (>400 nm, visible light, 300 W Xe lamp). For example, the CsPbBr_3_:*x*Li^+^ NCs show degradation of RhB dye in dichloromethane by 50% within 10 min of solar light exposure, which is superior compared to pure CsPbBr_3_ NCs (Figure S15, Supporting Information).

**Figure 4:**
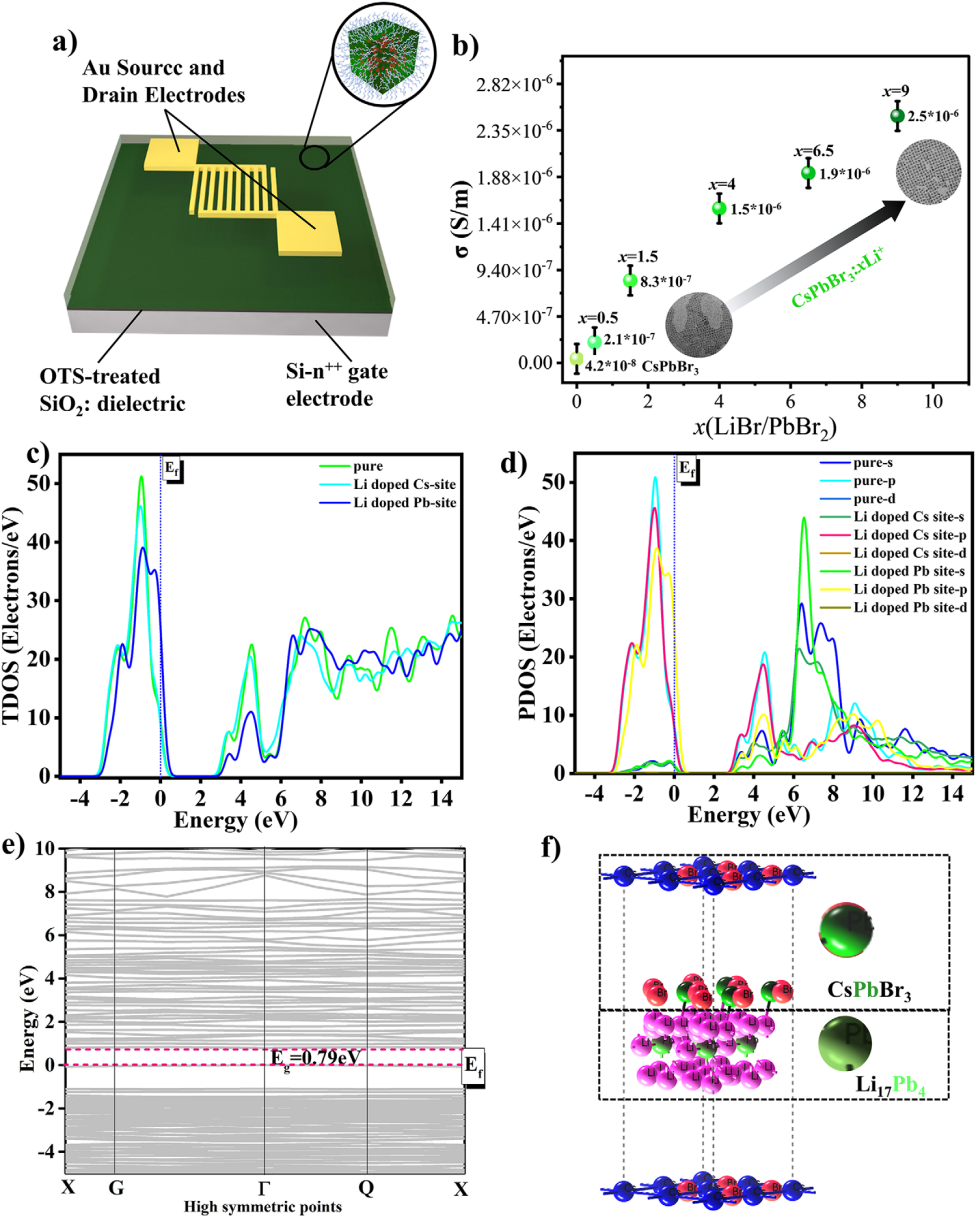
a) Structure of the field emission transistors (FETs) in bottom‐contact bottom‐gate architecture (b) The electrical conductivity of CsPbBr_3_:*x*Li^+^ NCs (*x* = 0.5 to 9) measured at inert atmosphere (c) and (d) show the total and partial density of states (DoS) of pure and doped systems. e) and f) show the bandgap value and the heterostructure of CsPbBr_3_‐Li_17_Pb_4_.

The green emitting CsPbBr_3_:*x*Li^+^ NCs were studied for their conducting properties using bottom‐contact bottom‐gate field effect transistors (**Figure** **4**a). These transistors were fabricated with a ≈1 µm drop‐cast layer of perovskites with a concentration uniformly maintained at 5 mg mL^−1^ and drop cast into the channel. The I_DS_ current was measured as a function of the gate voltage and compared with each other. Figure 4b shows the electrical conductivity as a function of the change in the composition of the CsPbBr_3_:*x*Li^+^ NCs (*x* = 0.5 to 9). Since exposing halide perovskites to air can have a significant influence on their conductivity,^[^ ^60^ ^]^ all measurements were conducted in a dry and oxygen‐free nitrogen atmosphere. The transistor containing the CsPbBr_3_:*x*Li^+^ NCs films showed a gradual increment in their conductivity from 2.1 × 10^−7^ S m^−1^ to 2.5 × 10^−6^ S m^−1^. From each sample and step increment from 4, 2, 1.2, and 1.3 times are noticed. The best conductivity of these CsPbBr_3_:*x*Li^+^ NCs films (*x* = 9) was found to be 12‐fold higher compared to the devise based on CsPbBr_3_:*x*Li^+^ NCs (*x* = 0.5). The material with the highest intrinsic electrical conductivity showed a 50‐fold increase compared to pure CsPbBr_3_ NCs.

The increase in the conductivity is attributed to the increase of Li^+^ concentration (0.15% and 0.35%) and the mechanistic shift from clustering within the lattice to surface passivation. For better understanding of the conductivity while doping Li^+^ into the CsPbBr_3_ lattice, DFT studies were performed. In Figure 4c,d, the partial density of states and total density of states on Li^+^‐doped CsPbBr_3_ NCs is shown providing further insight into the electronic properties. Understanding the state involvement of the CB and VB near the Cs^+^, Pb^2+^, and halogen atoms is crucial, especially when analyzing the electronic properties and the corresponding photocatalytic activity of the Li modified perovskite materials (Figure S16, Supporting Information). Figure 3c,d provides the total density of states (TDoS) of atomic orbitals and partial density of states (PDoS), respectively. Basically, the VB is constructed by the Pb 5p orbitals, with no contribution from the Br, whereas the conduction band is composed of both Pb and the bromine contributions. Upon doping, the peaks shifted toward the Fermi level due to the transition of free electrons from the VB to the CB, altering the energy range from the original values (for details see Figure S16, Supporting Information). However, Cs⁺ orbitals show minimal involvement near the Fermi level since they reside deep within the CB and VB, as shown in Figure S16a (Supporting Information). Regarding Pb^2+^ and Li⁺ atoms, the elemental partial density of states (EPDoS) of Pb atoms reveals that the highest peaks in the VB appear at 2.19 eV (pure‐p), −2.24 eV (4p‐Li⁺ at the Cs site), and −2.11 eV (5p‐Li⁺ at the Pb site). High‐energy free electrons transition from the VB (leaving holes) cross the Fermi level into the CB, creating a new state in the CB that enhances electronic conductivity. The TDoS show that the Li^+^ doped Pb site contribute more to the valence band to shift toward the CB and passing 0.13 eV beyond the original density of states (Figure 4c). The PDoS of all the orbital contribution shows that as a result of Li^+^doping at Pb^2+^, the Pb related p orbitals shifted toward the conduction band (yellow trace in Figure 4d) crossing beyond the Fermi energy leve, indicating highest contribution to the increased electrical conductivity upon Li^+^ doping.

Beyond that, there is a possibility of Li_m_Pb_n_ clustering inside the lattice, forming novel heterostructures or surface passivation of this alloy, which could also contribute to the stability and increased conductivity. Major changes in elemental composition were observed in the case of Pb and Cs ions. This indicates the presence of Li_m_Pb_n_ clusters that produce metal ion defects. Such metal ion defects because of Li_m_Pb_n_ clustering has lead to the increased conductivity in CsPbBr_3_:0.5Li^+^ NCs compared to pure CsPbBr_3_ NCs. Additionally, in the case of CsPbBr_3_:0.5Li^+^ NCs, the Li^+^ is inside the lattice and is more prone to the surface passivation of Li‐oleate or in the form of Li_m_Pb_n_ alloy‐CsPbBr_3_ heterostructures. Schematic of such a heterostructure model is shown in Figure 4f. The band‐gap of the heterostructure CsPbBr_3_‐LiPb is found to be 0.79 eV which resulted in an improved conductivity compared to the perovskite like Cs‐Pb‐Br structure (Figure 4e,f).

**Figure 5:**
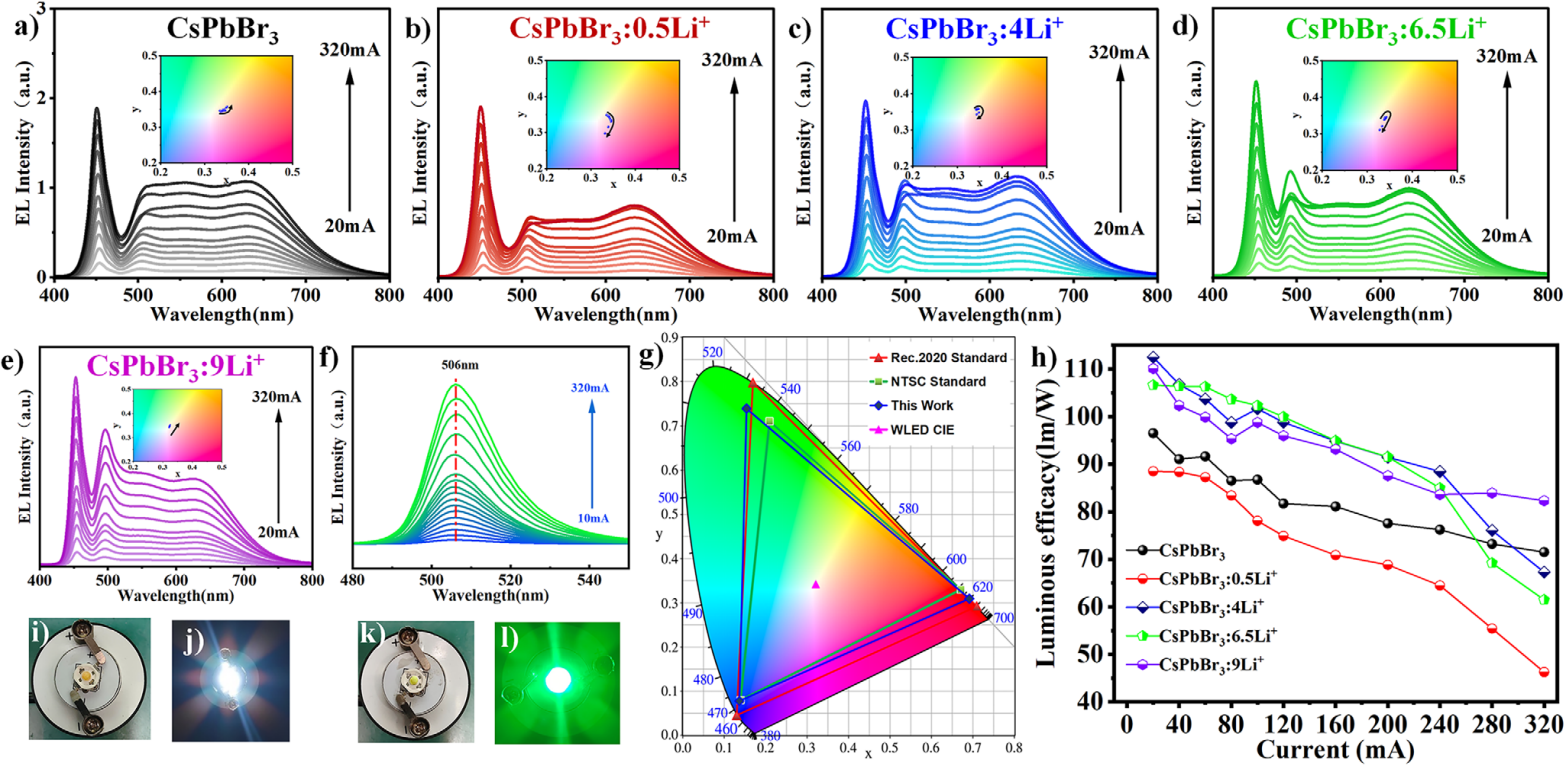
Emission spectra of white light‐emitting diodes (WLEDs) under different currents (from 20 mA to 320 mA): a) CsPbBr_3_, b) CsPbBr_3_:0.5Li^+^, c) CsPbBr_3_:4Li^+^, d) CsPbBr_3_:6.5Li^+^, and e) CsPbBr_3_:9Li^+^ f) Emission spectra of CsPbBr_3_:9Li^+^ of green light‐emitting diodes under different currents (from 10 to 320 mA). i) and j) display related photographs of the device and the working white light‐emitting diodes, while k) and l) display the related photographs of the device and the working green light‐emitting diodes. (g) Comparison of the color gamut of white light‐emitting diodes with the NTSC Television Standard and the Rec. 2020 Standard for CsPbBr_3_:9Li^+^. The CIE color coordinates of the white light‐emitting diode device at (0.32, 0.34). h) Luminous efficacy of each sample under white light‐emitting diodes operation at different currents (from 20 to 320 mA).

A white light emitting diode (WLED) device was fabricated by combining green CsPbBr_3_:*x*Li^+^ NCs, yellow‐green powder (Y_3_Al_5‐_ *_x_* Ga*_x_* O_12_:Ce^3+^) and red powder (CaAlSiN_3_:Eu^2+^) with a blue LED. This white LED device, designed for wide‐gamut applications, was achieved with relatively high thermal and operational stabilities. The result of electroluminescence (EL) spectrum of the WLED device is shown in **Figure** **5**. The EL spectrum is composed of four emission bands belonging to the blue LED chip (455 nm), our samples phosphor (near 500 nm), yellow‐green powder (550 nm) and red powder (640 nm). In Figure 5a–e, changes in EL intensity of CsPbBr_3_:*x*Li^+^ NCs were observed under different currents ranging from 20 mA to 320 mA. With the increase of drive current, the emission intensity of CsPbBr_3_:*x*Li^+^ (*x* = 0–9) NCs, showed an increasing trend with a gradual rise in current.

However, when the operating current reached 320 mA, the CIE color coordinates shifted significantly from initial white light to purplish‐pink luminescence region, due to the electrical quenching of the green light components (CsPbBr_3_ NCs) (Figure 5b–d) and their insets depicting changes in color coordinates). For example, in the case of CsPbBr_3_:0.5Li^+^, CsPbBr_3_:4Li^+^, and CsPbBr_3_:6.5Li^+^ there was a reduction in EL intensity at 520 nm under 320 mA current, hence, the brightness of the light intensity decreased toward the red region. In the case of CsPbBr_3_:0.5Li^+^, the initial coordinates (0.336, 0.3488) shifted to (0.3315, 0.2984). The inset shows reduced brightness compared to the bright white light (Inset of Figure 5b). The other two compositions also showed similar attributes as shown in the insets of Figure 5c,d. This could be attributed to the Li^+^ ion migration within the lattice, which could destabilize the structures and eventually reduce EL properties. Note that, from the XPS results of CsPbBr_3_:xLi^+^, where *x* = 0.5, the Li^+^ ions are integrated into the lattice making them prone to migration within the lattice. Such reduction in EL intensity is not noticeable in the case of pure CsPbBr_3_ NCs. In contrast, for the sample CsPbBr_3_:9Li^+^, the EL showed an increasing trend, indicating that their structures could be better maintained under high currents, supported by the presence of Li^+^ on their surface. Moreover, within the same EL range, the value of the sample with CsPbBr_3_:9Li^+^ showed a significant upward trend, indicating better stability. As discussed previously, in these NCs, most of the Li^+^ remained on the outer surface instead of being within the core. The Li‐oleate outer layer helps to protect the NCs surface and further enhances their stability. Such layer passivation is also noticeable in the case of K‐oleate and Na‐oleate surface‐passivated CsPbBr_3_ NCs.^[^ ^37^ ^]^ The order of the luminescence efficacy values at 20 mA for pure CsPbBr_3_ and CsPbBr_3_:*x*Li^+^ NCs (*x* = 0 to 9) is: 96.5, 88.5, 112.5, 106.6, and 110.1 lm/W. The quantum yield values for these samples are 50, 45, 67, 65, 64%, respectively. The PLQY values for the last three compositions (*x* = 4 to 9) are similar and hence, the luminescence efficacy values are close to each other (Figure 5h).

Among them, the highest efficacy value (112.5 lm W^−1^) corresponds to CsPbBr_3_:4Li^+^ NCs. However, this sample undergoes structural distortion upon increase in the current. Hence, it is not considered as an ideal material in terms of its luminescence efficacy beyond 240 mA (Figure 5h, blue trace). Similar trends were also observed in the case of composition CsPbBr_3_:6.5Li^+^ (Table S8, Supporting Information). For comparison, the state of the halide perovskite based materials developed along with their Luminous efficacy values were summarized in Table S9 (Supporting Information). The values of the specific reaction rate constants for the dye degradation were shown in Table S7 (Supporting Information). The constructed WLED device for CsPbBr_3_:9Li^+^ NCs exhibited the CIE color coordinates (0.321,0.342) (Figure 5e), a color temperature of 6011 K, and an efficiency of 110.31 lm/W. The obtained color gamut of the assembled lighting display is compared with the NTSC and Rec.2020 standards (Figure 5g). The color gamut reported in this work can cover ≈ 114.2% and 85.2% of the NTSC and Rec.2020, respectively. The corresponding values for each of the samples were shown in Tables S10 and S11 (Supporting Information). In this case, CsPbBr_3_:9Li^+^ NCs, the Li remaining on the outer shell of the NCs, helps to protect them against increased currents, and the device is stable at various current values. The Ra values of these materials at various current values are presented in Tables S12–S16 (Supporting Information). These values are in agreement with the previously reported color rendering index, R,a values of CsPbBr_3_ NCs.^[^ ^66^ ^]^

The fabricated green LEDs with color coordinates (0.0557, 0.7113) using these mixed‐cation CsPbBr_3_:9Li^+^ NCs. Figure S17 (Supporting Information) shows the evolution of EL spectra of LEDs with different injection current (Figure 5f), in which the peak position of EL spectra did not change during the testing procedure, which is attributed to the constant Stark effect. The solution phase and EL spectra are comparable without much shift in their PL wavelength. The intensity of the pure green light gradually increased for green LEDs as the driving current increased from 10 to 320 mA. The solution PL is around 498 nm, which shifted by 8 nm further. Such a slight shift was attributed to the fact that NCs are self‐assembled in the solid state, unlike in solution, where the particles are isolated. However, the emission peak position (507 nm) remained constant during these current increments, indicating that the sample exhibited excellent stability even under higher currents. In contrast, coated LEDs exhibited robust stabilities even under a high injection current (i.e., the emission peak remained at the same position), as shown in Figure 5f. These results demonstrate that these CsPbBr_3_:9Li^+^ NCs are stable and represent a useful strategy to improve the performance of green LED devices.

## 3 Conclusion

Significant improvements in both electrical conductivity and optical properties of CsPbBr_3_ NCs through the interaction with Li⁺ ions is reported. Successful doping of Li⁺ into CsPbBr_3_ NCs is achieved owing to the novel synthetic procedure adopted. Apart from Li^+^ doping the perovskite lattice, formation of Li_m_Pb_n_ type alloy species was observed which has lead to the surface passivation, and contributed up to a 50‐fold increase in conductivity compared to pure CsPbBr_3_ NCs. Such modification of chemical composition, structure and morphology, has resulted in a significant enhancement in photoluminescence quantum yield (PLQY), rising from 50% to 67% and an overall device performance. The degree of lattice insertion is finely tuned by the ratio of LiBr to PbBr_2_ during synthesis using the hot injection method. Additionally, hydrolyzed LiBr induces a phase transition to Cs_4_PbBr_6_, which exhibits weaker interactions with Li^+^, highlighting the role of surface and lattice interactions in property tuning. In white LED applications, CsPbBr_3_:xLi NCs resulted in the enhancement of luminous efficiencies from 88.5 to 112.5 lm W^−1^, surpassing the performance of pure CsPbBr_3_ NCs. Density functional theory (DFT) calculations suggest that electron movement from the conduction band to the valence band further boosts conductivity, while the formation of Li_m_Pb_n_ alloy species and surface passivation likely reduces the cation vacancies, contributing to improved performance These findings offer valuable insights into doping strategies and the potential of CsPbBr_3_:xLi^+^ ‐ Li_m_Pb_n_ heterostructures, paving the way for advanced optoelectronic applications that benefit from enhanced conductivity and luminous efficiency. There is still large scope for improving the PLQY by optimizing various stages in the preparation of the Li modified halide perovskite systems.^[^ ^67^ ^]^

## Conflict of Interest

The authors declare no conflict of interest.

## Author Contributions

T.U. conceived the idea and supervised the research. Z.G. performed synthesis, optical, and morphological characterizations of the perovskites. M.P., S.A.M., H.R.H., and L.D., assisted TEM characterizations, and analyzed the data. S.W., Z.W., and T.L. performed the photo FET fabrication and characterizations. M.M. performed DFT calculations. T.U., Z.G., and T.L. wrote the paper. All authors discussed the contents and contributed to the manuscript.

## Supporting information



Supporting Information

## Data Availability

The data that support the findings of this study are available from the corresponding author upon reasonable request.
