# Supplementary material for: Boosting Electronic Properties of CsPbBr3 Nanocrystals via Lithium‐Ion Doping and Surface Passivation for Enhanced Electrical Conductivity and Efficient White Light‐Emitting Diodes
Source: Adv Sci (Weinh). 2025 Jun 5;12(28):2417304. doi: 10.1002/advs.202417304 (PMC12302538; doi:10.1002/advs.202417304)
Supplement: Supplementary file 1 — Supporting Information [file ADVS-12-2417304-s001.docx]

**Supporting information**

Boosting Electronic Properties of CsPbBr_3_ Nanocrystals via Lithium-Ion doping and Surface Passivation for enhanced electrical conductivity and Efficient White Light-Emitting Diodes

Zhongsheng Ge,^1^ Siyuan Wan,^1^ Muhammad Moin,^1^ Sk Abdul Moyez,^1^ Lizhuang Dong,^1^ Hamood ur Rehman Haris,^1^ Marek Piotrowski,^1^ Zhiming Wang,^1^ Tim Leydecker,^1，*^ Udayabhaskararao Thumu^1，*^

^1^Institute of Fundamental and Frontier Sciences, University of Electronic Science and Technology of China, Chengdu 610054, China.

Email: tim_leydecker@uestc.edu.cn; uday@uestc.edu.cn.

**Supporting information S1**

***Experimental section***

***Materials***

1-octadecene (ODE, 90%), oleic acid (OA, 90%), cesium carbonate (Cs_2_CO_3_ ,99.9%) and dichloromethane were purchased from Aladdin. Methyl acetate (MeOAc, 99.5%) and hexane (97%) were purchased from General-Reagent. Oleylamine (OLAM, 90%) were purchased from Heowns, Chengdu, China. Lead bromide (PbBr_2_, 99.9%) and lithium bromide (LiBr) were purchased from Meryer, Chengdu, China.

***Synthesis of Nanocrystals (NCs)***

**Cesium oleate synthesis:** Cs_2_CO_3_ (0.814 g) was placed in a 100 mL three-neck flask along with octadecene (40 mL) and oleic acid (2.5 mL). The mixture was dried under vacuum at 120 °C for 1 hour. Subsequently, it was heated under a nitrogen atmosphere to 150 °C, ensuring complete reaction of Cs_2_CO_3_ with oleic acid, as indicated by the absence of white precipitate at the bottom of the flask. Upon cooling to room temperature, the solution transformed into a white solid.

**Synthesis of pure CsPbBr_3_ NCs:** In octadecane (5 mL), 0.069 g of PbBr_2_ was taken in 25 mL three- necked round-bottom flask and heated at 120 °C under vacuum for 45 minutes. Next, the vacuum was switched to inert gas, and oleic acid (OA, 0.5 mL) and oleylamine (OLAM, 0.5 mL) were injected into the above solution. At this stage the PbBr_2_ dissolved due to the formation of lead oleate and oleylamine bromide species, leading to a clear yellow colored solution. Then, pre-heated cesium oleate solution was injected into the solution resulting the formation of CsPbBr_3_ NCs, observed by visible changes in the solution color from pale yellow to bright green. This solution was cooled down using ice-cold conditions within 10 seconds of their formation.

**Synthesis of CsPbBr_3_:Li^+^ NCs at dry and wet conditions:** For the synthesis of CsPbBr_3_:Li^+^NCs, the procedure followed was the same as above, except the starting precursors included both PbBr_2_ and LiBr (LiBr was weighed under Ar atmosphere in a glove box to avoid exposure to moisture). The ratios of PbBr_2_:LiBr were varied as given in the Table S1 below. The same PbBr_2_:LiBr ratios were used for experiments conducted under hydrolyzed conditions (Table S2). In this case, first LiBr and PbBr_2_ were added to a RB flask, then 5 μL of water was introduced, followed by the addition of ODE and application of vacuum for 45 minutes. The remaining procedure followed as per the above procedure. The as synthesized NC solution, after reaching the room temperature, was subjected to centrifugation at 3500 rpm for 10 minutes to remove unreacted salts (and some cases the Li_m_Pb_n_ alloy complexes). The NCs present in the supernatant was precipitated by methyl acetate to remove the excess ligands or unreacted precursors. The purified CsPbBr_3_:*x*Li^+^ NCs or Cs_4_PbBr_6_:*x_w_*Li^+^ NCs were dispersed in hexane for further in characterization. Here, *x* represents the LiBr concentrations with respect to PbBr_2_ at dry conditions and the *x_w_* represent the LiBr concentrations with respect to PbBr_2_ at wet conditions. The Cs_4_PbBr_6_:*x_w_*Li^+^ NCs are formed only in the presence of water at higher LiBr concentrations (Specific addition amounts are shown in **Table S1 and S2**).

**PLQY measurements:**

Photoluminescence (PL) measurements were conducted using Rhodamine B (RHB) as a reference standard. The CsPbBr_3_:*x*Li^+^ NCs exhibited PLQY below 70%, lower than literature reports (>90%), primarily due to multiple washing steps performed using methyl acetate to remove the precursors or unwanted Li_m_Pb_n_ complexes. During this process, the partial removal of surface ligands (such as oleic acid and oleylamine) led to an increase in non-radiative recombination centres. The polar solvent also induced minor NC aggregation, further reducing emission efficiency.

**Table S1.** Reaction conducted at dry conditions, with all the reactions conducted at 120 °C.

| CsPbBr_3_:*x*Li^+^ NCs | Amount of PbBr_2_ (mg) | Amount of LiBr (mg) |
| --- | --- | --- |
| 1. CsPbBr_3_:0.5Li^+^ NCs | 69 | 8.2 |
| 1. CsPbBr_3_:1.5Li^+^ NCs | 69 | 24.5 |
| 1. CsPbBr_3_:4Li^+^ NCs | 69 | 65.3 |
| 1. CsPbBr_3_:6.5Li^+^ NCs | 69 | 106.1 |
| 1. CsPbBr_3_:9Li^+^ NCs | 69 | 146.9 |

**Table S2.** Reaction conducted at wet conditions at 120 °C.

| CsPbBr_3_:*x_w_*Li^+^ NCs | Water (μL) | Amount of PbBr_2_ (mg) | Amount of LiBr (mg) |
| --- | --- | --- | --- |
| 1. CsPbBr_3_:0.5_w_Li^+^ NCs | 5 | 69 | 8.2 |
| 1. CsPbBr_3_:1.5_w_Li^+^ NCs | 5 | 69 | 24.5 |
| 1. CsPbBr_3_:4_w_Li^+^ NCs | 5 | 69 | 65.3 |
| 1. Cs_4_PbBr_6_:6.5_w_Li^+^ NCs | 5 | 69 | 106.1 |
| 1. Cs_4_PbBr_6_:9_w_Li^+^ NCs | 5 | 69 | 146.9 |

**Synthesis of Li-OA/OLAM, Pb-OA/ OLAM, and LiPb-OA/ OLAM complexes:** LiBr (0.106 g), PbBr_2_ (0.202 g), and equal amounts of LiBr and PbBr_2_ (0.069 g each) were placed separately into three 25 mL three-neck flasks. To each flask, 5 mL of octadecene (ODE) was added, and the mixtures were dried under vacuum at 120 °C for 1 hour. Subsequently, oleylamine (OLAM, 0.5 mL) and oleic acid (OA, 0.5 mL) were injected at 120 °C under a nitrogen atmosphere. After allowing the reaction to proceed, the three mixtures were stored in a refrigerator for use at a later time.

***Characterization***

Transmission electron microscope (TEM), high-resolution transmission electron microscope (HRTEM) images were collected on a 200 kV TECNAI G2 F20 with a Gatan SC200 CCD camera from FEI, USA. Energy-dispersive X-ray spectroscopy (EDS) in high-angle annular dark field scanning TEM mode (HAADF-STEM-EDS) studies were performed on the same 200 kV TECNAI G2 F20, FEI, USA. X-ray diffraction (XRD) patterns were measured on a desktop diffractometer (D2 PHASER, Bruker, Germany) with a Cu Kα source. All absorption spectra were recorded in a range of 200-800 nm using a UV spectrometer (SPECORD S600). Photoluminescence (PL) measurements were carried out using a fluorescence spectrophotometer (FLUOROMAX-4). Photoluminescence quantum yield (PLQY) and lifetime measurements were collected using the fluorescence spectrophotometer (FLS-1000). LED optoelectronic testing was collected on OHSP-350M LED Fast-Scan Spectrophotometer（350-1050 nm）from Hangzhou Hopoo Light & Color Technology Co., Ltd. X-ray photoelectron spectroscopy (XPS) patterns were measured using the Thermo Scientific K-Alpha instrument from the United States with an excitation source of Al Kα radiation (hv = 1486.6 eV). The elemental composition of the resulting solution was then accurately measured using an ICP-MS instrument (PerkinElmer NexION 300X).

***LED assembly***

For the fabrication of green emitting NCs devices, CsPbBr_3_:*x_w_*Li^+^NCs powder was mixed in a mass ratio of 1:1 between powder and UV curing adhesive. The resulting semi-solid sample was placed on the 3W LED chip with a wavelength of approximately 460-465 nm and is provided by Sanan Optoelectronics Co., Ltd. Subsequently, tests were carried out with the current varying from 10 mA to 320 mA to obtain the electroluminescence spectra.

The fabrication process of white light emitting NCs devices involved adding CsPbBr_3_:*x*Li^+^ NCs powder, and commercial yellow-green powder (Y_3_Al_5-x_Ga_x_O_12_:Ce^3+^, with a main peak wavelength of 550 nm, provided by Yantai Hilde new materials, Chengdu, China), and nitride red powder (CaAlSiN_3_:Eu^2+^, with a main peak wavelength of 640 nm, from Intematix Co., Ltd., USA). These powders were mixed in an approximate mass ratio of sample powder, green powder, and red powder of 1:5:5. Finally, UV curable adhesive was used to blend the powder and adhesive in a 1:1 mass ratio (referring to the total mass of all powder mixtures). The LED chip utilized was 3W, with a wavelength of approximately 455 nm and was also provided by Sanan Optoelectronics Co., Ltd. Xiamen, Fujian. Subsequently, tests were carried out with the current varying from 10 mA to 320 mA to obtain the white light emitting electroluminescence (EL) spectra.

***DFT computational details***

The structural parameters and electronic responses of pure and Li-doped Cs_1-x_Li_x_PbBr_3_ and CsPb_1-x_Li_x_Br_3_ perovskite materials were investigated using the plane-wave pseudopotential method within the framework of Density Functional Theory (DFT). This approach was implemented using the CASTEP module in Materials Studio, which offers a reliable platform for analyzing atomic-level responses of materials. A plane-wave cut-off energy of 520 eV was applied during the calculations. To sample the Brillouin Zone accurately, a (12 × 12 × 12) Monkhorst-Pack k-point mesh was used. The primary goal was to optimize the geometry of the crystal structure, for which convergence thresholds were set as follows: a total energy convergence of 2 × 10^-5^ eV/atom, a maximum force convergence of 0.03 eV/Å, a maximum stress convergence of 0.05 GPa, and a maximum displacement convergence of 1 × 10^-3^ Å. These criteria ensured the accurate determination of the optimized crystal structure. To investigate the electronic properties and band gaps, crucial for evaluating the potential of these materials in photovoltaic applications, the generalized gradient approximation (GGA) using the Perdew-Burke-Ernzerhof (PBE) functional simulation study was employed. This methodology provided detailed insights into band gap engineering and the electronic structure of both pristine and doped Cs_1-x_Li_x_PbBr_3_ and CsPb_1-x_Li_x_Br_3_.

***Fabrication of FET-type device and conductive measurements***

**Device fabrication:** Interdigitated bottom-contact bottom-gate configuration transistors consisted of n^++^-Si substrates with 200 nm of thermally grown SiO_2_ as the gate dielectric and patterned pairs of gold electrodes with interdigitated geometry as the source and drain. All solutions, samples and devices were prepared and measured in a nitrogen filled glovebox to avoid oxidative doping and degradation of the materials and ensure reproducibility of the experiments. Pure CsPbBr_3_ NCs and CsPbBr_3_:*x*Li^+^ NCs perovskite samples were fabricated by deposition via drop-casting of 100 µL of a 5 mg/mL perovskite solution. All the solutions were prepared using hexane as solvent.

**Device characterization:** Intrinsic electrical conductivity of NCs were evaluated using a Keithley 2636B source meter. All electrical characterizations were performed in the glove box under nitrogen environment.


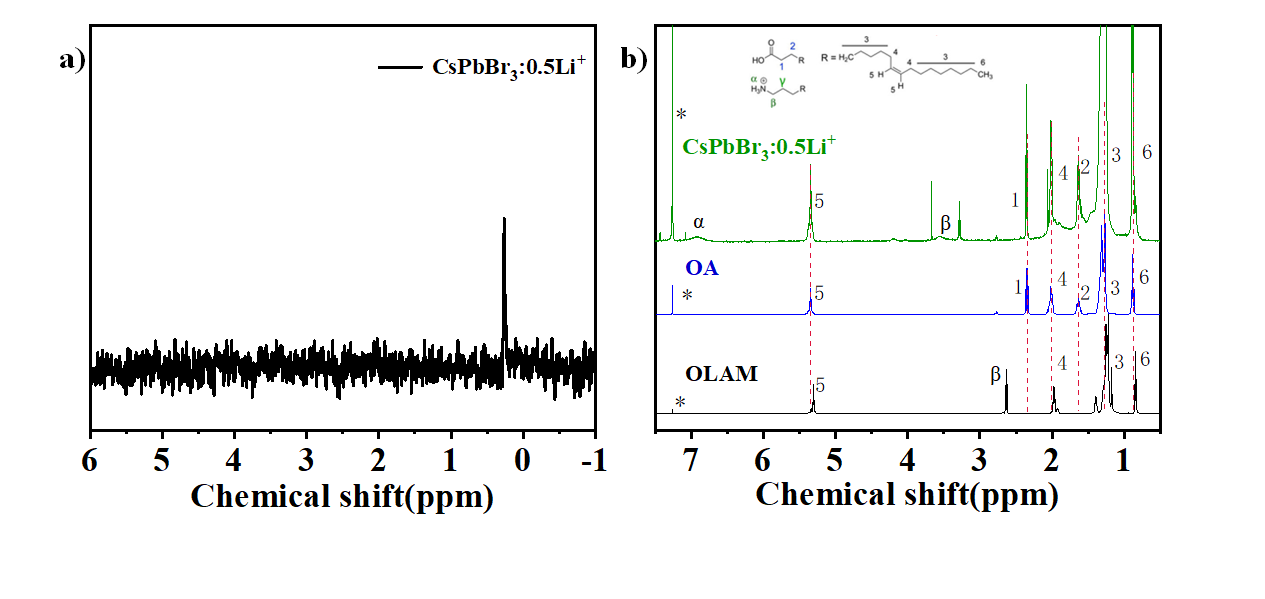


**Figure S1.** Figure a displays the ^7^Li NMR of CsPbBr_3_: 0.5 Li^+^, while Figure b shows the ^1^H NMR of oleylamine (OLAM), oleic acid (OA) and CsPbBr_3_: 0.5 Li^+^.

**
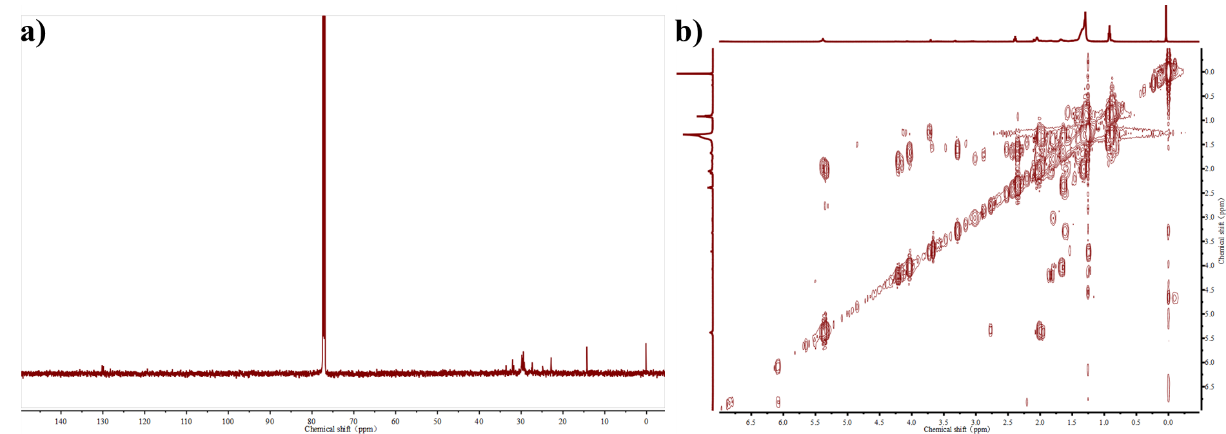
**

**Figure S2.** Figure a and b shows the ^13^C NMR and COSY NMR spectrum of CsPbBr_3_: 0.5 Li^+^.


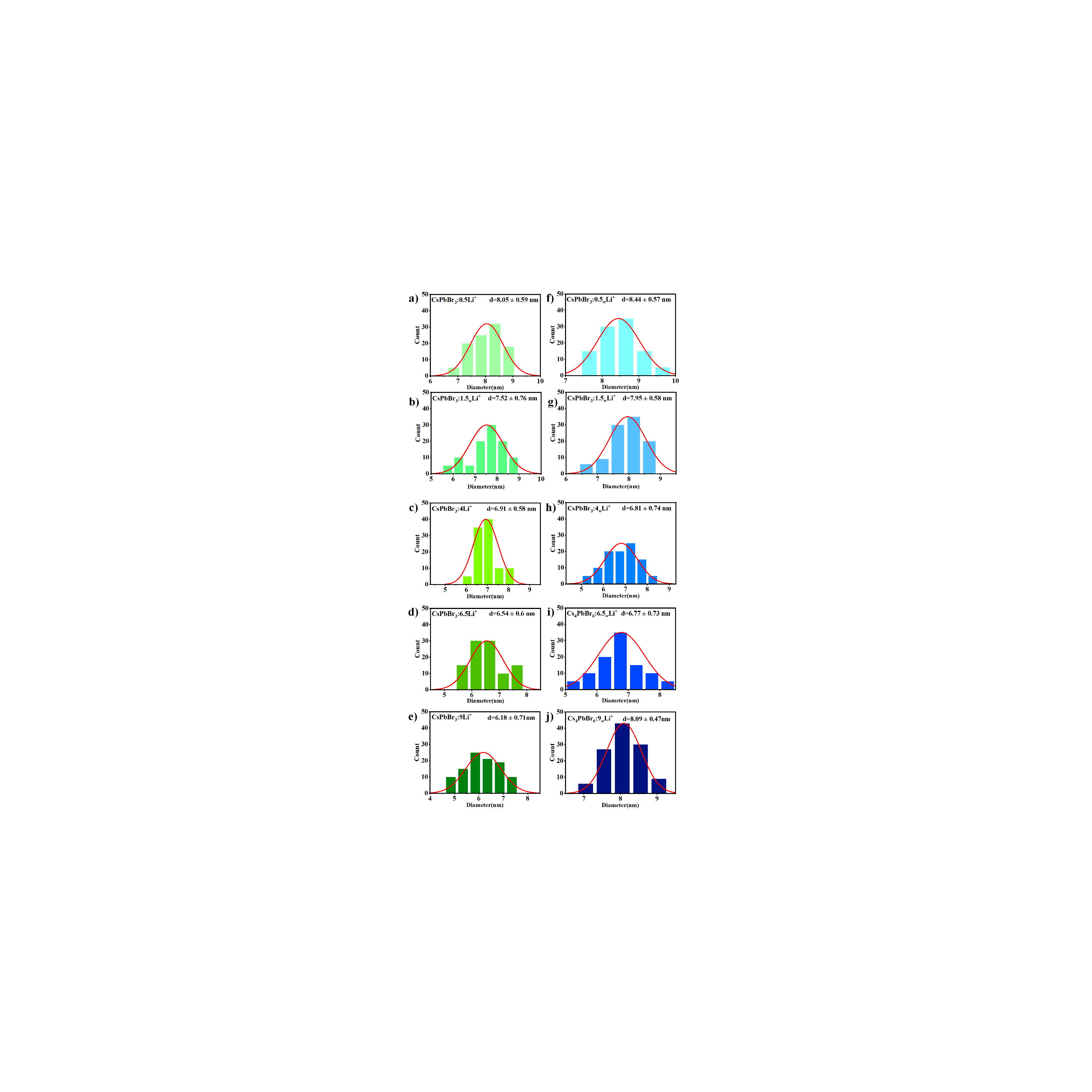


**Figure S3.** The size distributions are obtained from the TEM analysis, CsPbBr_3_:*x*Li^+^ NCs (*x*=0.5, 1.5, 4, 6.5, 9) (a-e), CsPbBr_3_:*x_w_*Li^+^ NCs (*x*=0.5, 1.5, 4) (f-h) and Cs_4_PbBr_6_:*x_w_*Li^+^ NCs (*x*=6.5, 9) (i-j).

**Figure S4.** STEM images of CsPbBr_3_:4_w_Li^+^ NCs (a) and Cs_4_PbBr_6_:9*_w_*Li^+^ NCs (b).

**Table S3.** The FWHM values of the PL spectra.

| Sample | FWHM (nm) | Sample | FWHM (nm) |
| --- | --- | --- | --- |
| CsPbBr_3_:0.5 Li^+^ | 25 | CsPbBr_3_:0.5*_w_*Li^+^ NCs | 23 |
| CsPbBr_3_:1.5Li^+^ NCs | 22 | CsPbBr_3_:1.5*_w_*Li^+^ NCs | 23 |
| CsPbBr_3_:4Li^+^ NCs | 22 | CsPbBr_3_:4*_w_*Li^+^ NCs | 23 |
| CsPbBr_3_:6.5Li^+^ NCs | 24 | Cs_4_PbBr_6_:6.5*_w_*Li^+^ NCs | 26 |
| CsPbBr_3_:9Li^+^ NCs | 21 | Cs_4_PbBr_6_:9*_w_*Li^+^ NCs | 27 |

**Figure. S5** STEM-EDS analysis showing the presence of Cs-L, Pb-L, Br-L mapping, along with their spectra, for CsPbBr_3_:4Li^+^NCs (a), CsPbBr_3_:9Li^+^NCs (b), CsPbBr_3_:4*_w_*Li^+^NCs (c) and Cs_4_PbBr_6_:9*_w_*Li^+^NCs (d). The atomic ratios are 1:1:3, 1:1:3, 1:1:3 and 4:1:6 for cases of (a), (b), (c), and (d), respectively. This clearly suggests that their respective compositions are CsPbBr_3_ and Cs_4_PbBr_6_. Note that due to its low atomic weight, Li could not be detected in these EDS analyses.

**
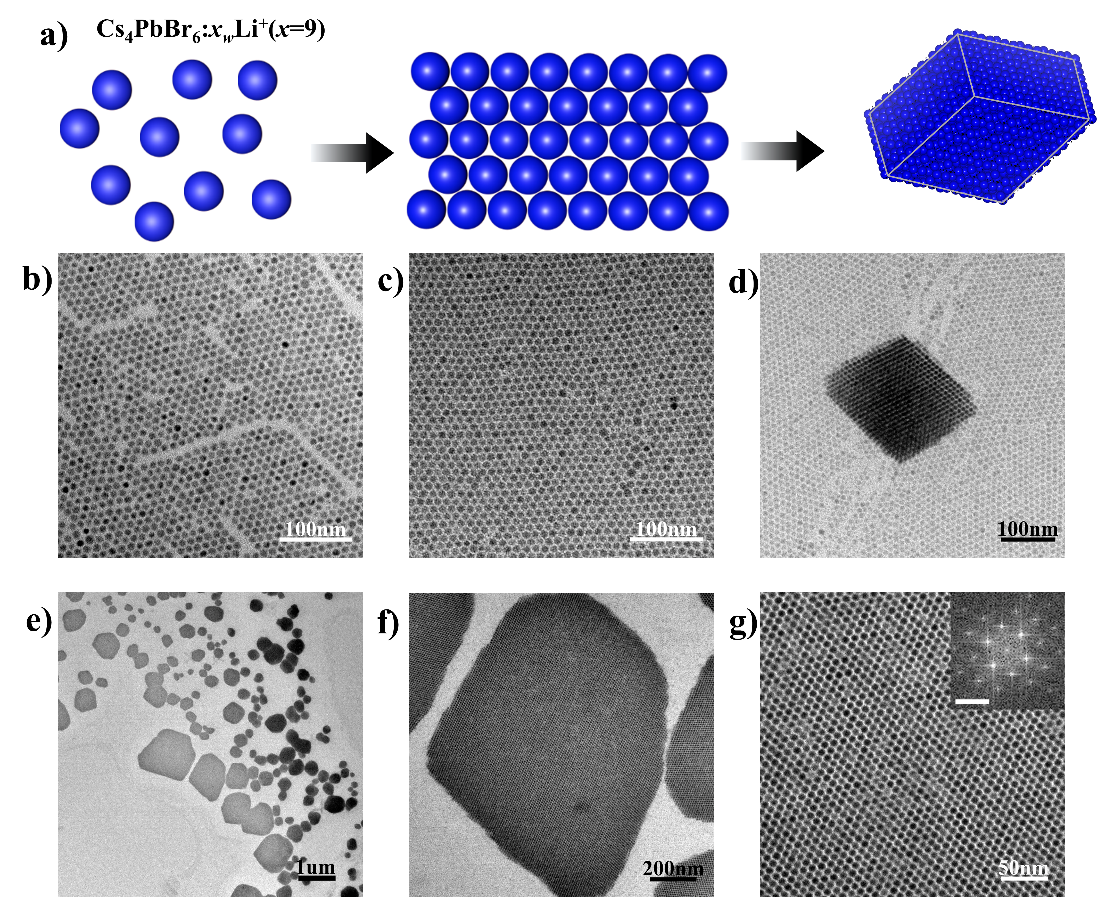
**

**Figure S6.** CsPbBr_3_:*x*Li^+^ NCs are spontaneously self-assembled into their well-organized superlattice structures, as schematically represented in (a). (b-d) show the initial process of self-assembly (2D and 3D growth). (e-g) show the 3D supercrystals at different scales.

**
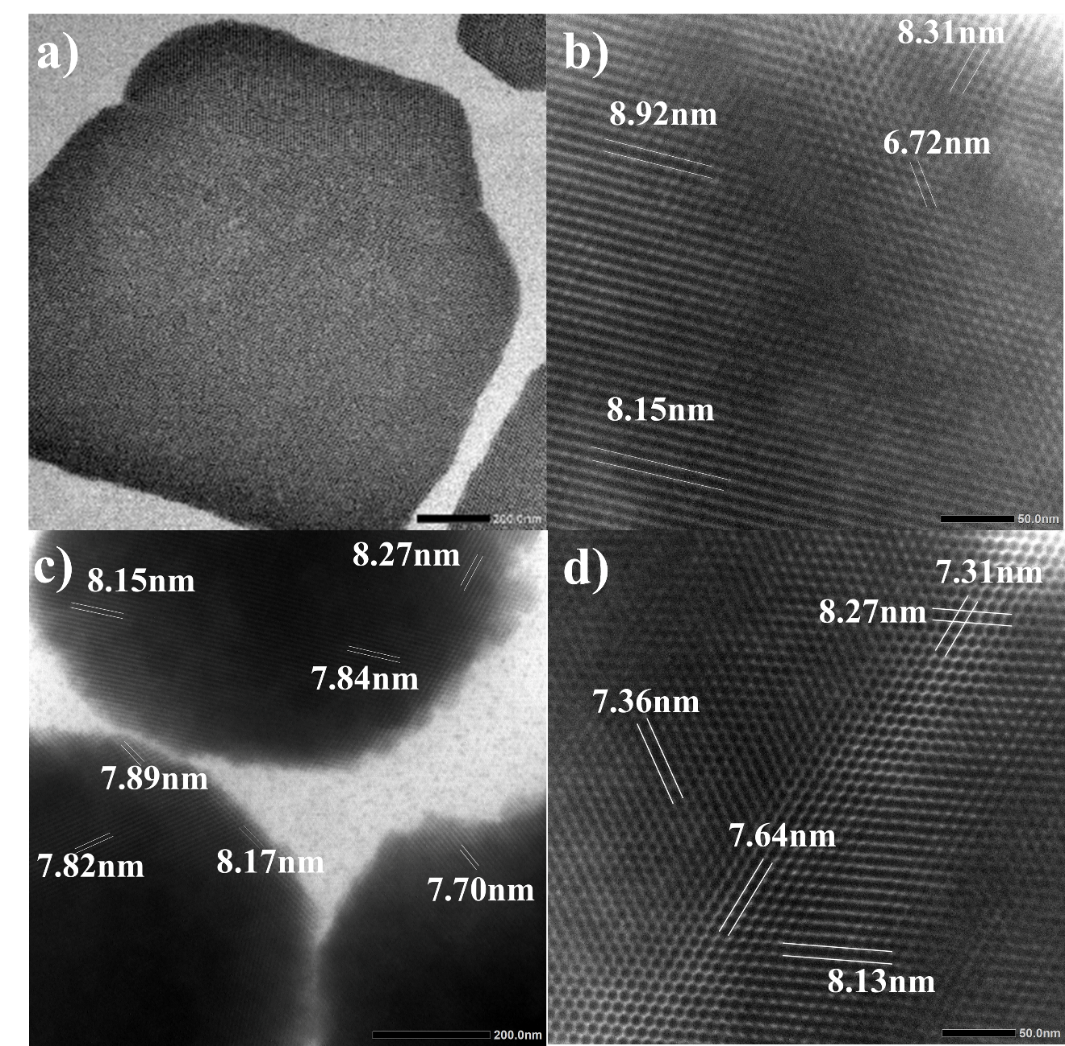
**

**Figure S7.** TEM images of the self-assembled structure of Cs_4_PbBr_6_:9_w_Li^+^ NCs. The NCs sizes are 7.5-8.9 nm. The inter-particle separation was smaller than the theoretical values of OA and OLAM (2.2 nm).


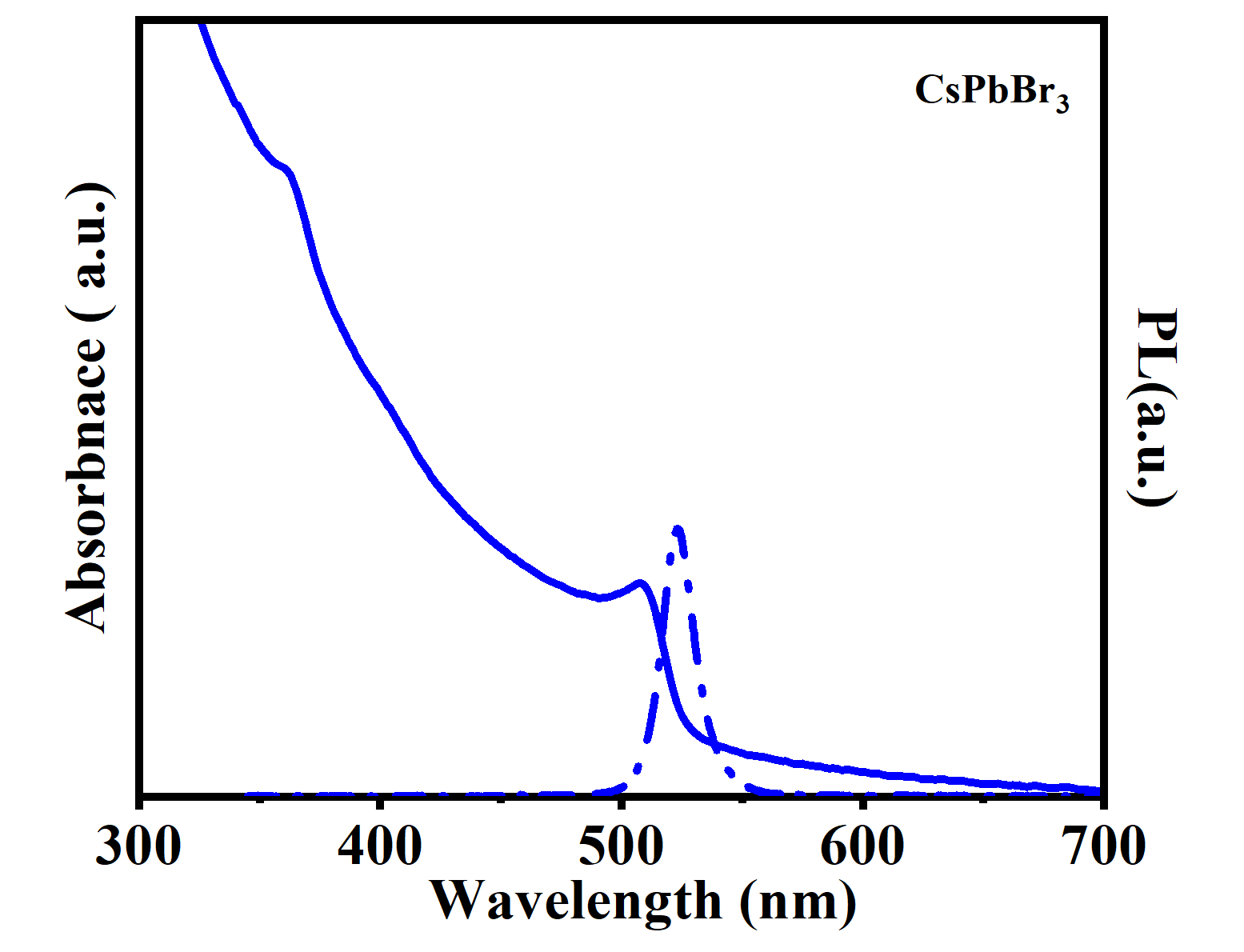


**Figure S8.** The absorption spectra and PL emission of the pure CsPbBr_3_ NCs (absorption and emission maxima for pure CsPbBr3 NCs observed at 510 nm and 524 nm)

**Table S4.** Analysis of Li to Cs ratio from XPS analysis of Li 1*s* and Cs 3*d* peak areas.

| Type of NCs | Li | Cs | Li:Cs |
| --- | --- | --- | --- |
| CsPbBr_3_:4Li^+^ NCs | 81.3 | 53066.6 | 0.0015:1 |
| CsPbBr_3_:4*_w_*Li^+^ NCs | 242.5 | 129199.2 | 0.0019:1 |
| CsPbBr_3_:9Li^+^ NCs | 225.2 | 62852.7 | 0.0036:1 |
| Cs_4_PbBr_6_:9*_w_*Li^+^ NCs | 124.8 | 171792.3 | 0.0007:1 |

**Table S5.** The percentages of metal ion doping into CsPbBr_3_ perovskites from the literature [1-6].

| Dopant | Dopant amount (%) | Reference |
| --- | --- | --- |
| Bi^3+^ | 0.25 - 2.1 (mol %) | [1] |
| Ca^2+^ | 13.09 (mol %) | [2] |
| Rb+ | 19.14 (atom %) | [3] |
| Cu^2+^ | 2.85 and 4.18 (atom %) | [4] |
| Cd^2+^, Zn^2+^, Sn^2+^ | 3.89, 1.64, and 2.77 (atom %) respectively | [5] |
| Fe^2+^ | 10 - 50 (atom %) | [6] |
| Li^+^ | 0.06 - 0.02 (wt. %) | Our work |

**Table S6.** ICP-MS data on the amount of Li, Cs and Pb in the samples CsPbBr_3_:0.5Li⁺, CsPbBr_3_:4Li⁺, CsPbBr_3_:6.5Li⁺, CsPbBr_3_:9Li⁺, and Cs_4_PbBr_6_:6.5Li⁺.

| **sample** | **Li (wt %)** | **Cs (wt %)** | **Pb (wt %)** |
| --- | --- | --- | --- |
| CsPbBr_3_:0.5Li⁺ | 0.02622 | 7.12123 | 12.7909 |
| CsPbBr_3_:4Li⁺ | 0.021546 | 11.3859 | 23.5939 |
| CsPbBr_3_:6.5Li⁺ | 0.061063 | 13.0552 | 31.7141 |
| CsPbBr_3_:9Li⁺ | 0.0640777 | 4.8893 | 9.812 |
| Cs_4_PbBr_6_:6.5Li⁺ | 0.0225487 | 3.71947 | 7.23813 |

**
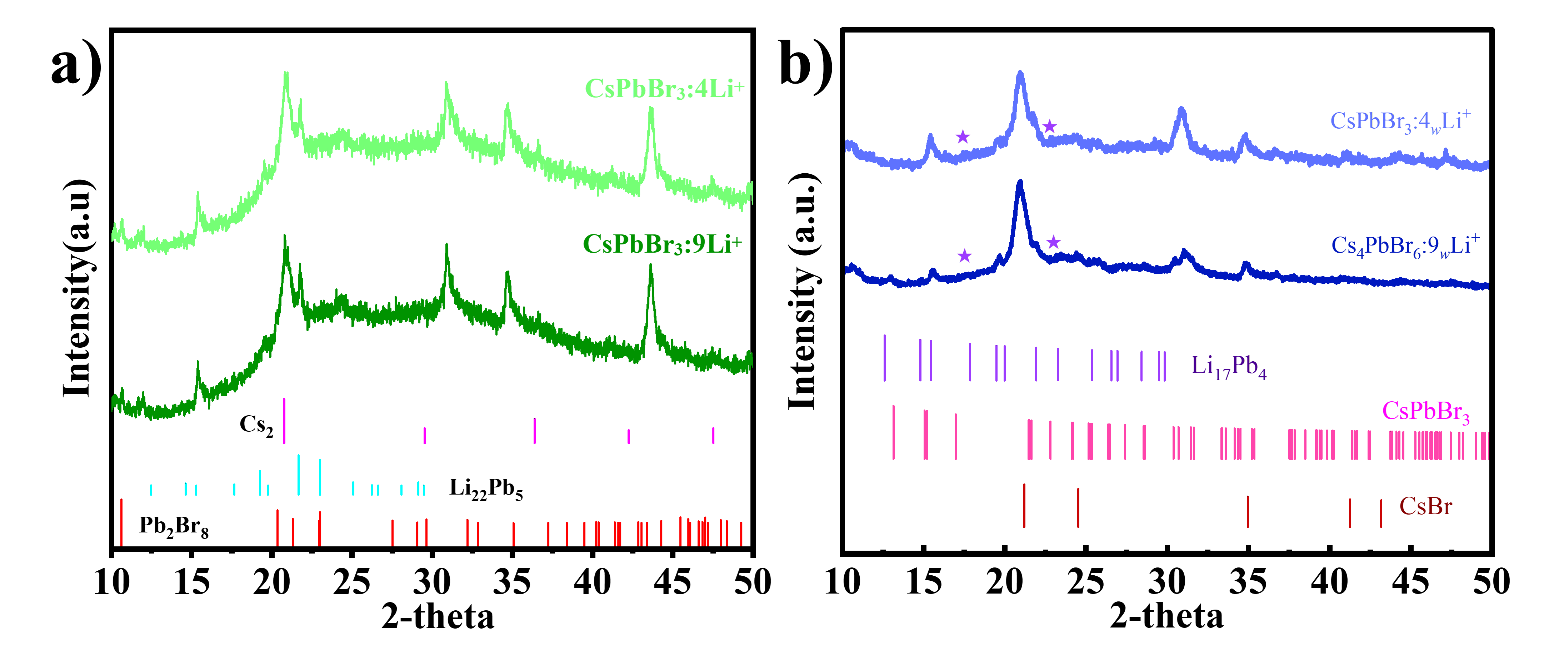
**

**Figure S9.** The XRD spectra of the precipitate showing the presence of Li_m_Pb_n_ alloy, received upon the first centrifugation of the as-synthesized NC solutions, namely CsPbBr_3_:4Li^+^ NCs and CsPbBr_3_:9Li^+^ NCs (a), and CsPbBr_3_:4*_w_*Li^+^ NCs and Cs_4_PbBr_6_:9*_w_*Li^+^ NCs (b). The cards used therein are Li_17_Pb_4_ #31-0687, CsPbBr_3_ #72-7929, CsBr #78-0615, Pb_2_Br_8_ (Cambridge Crystallographic Data Centre database code: 4319140), Cs_2_ (Cambridge Crystallographic Data Centre database code: 9011010), and Li_22_Pb_5_ (Inorganic Crystal Structure Database collection code: 104766).


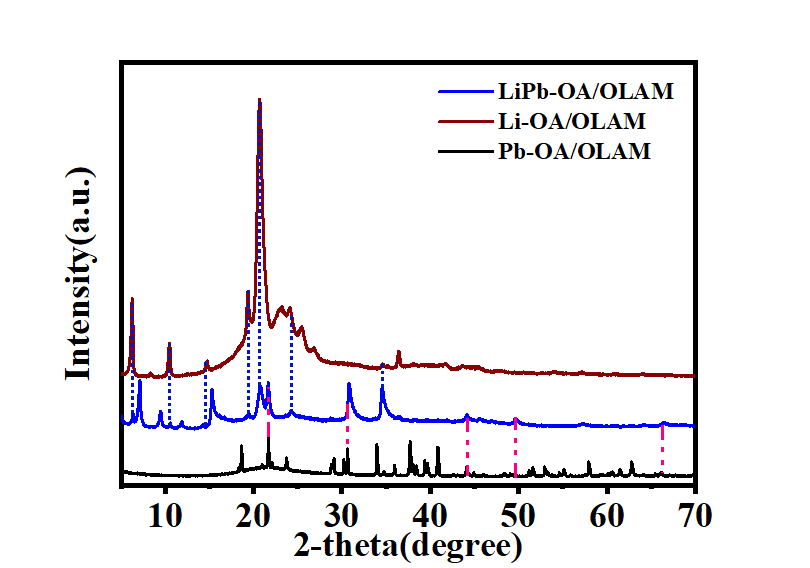


**Figure S10.** XRD patterns of Li-OA/OLAM, Pb-OA/OLAM, and LiPb-OA/OLAM.

**
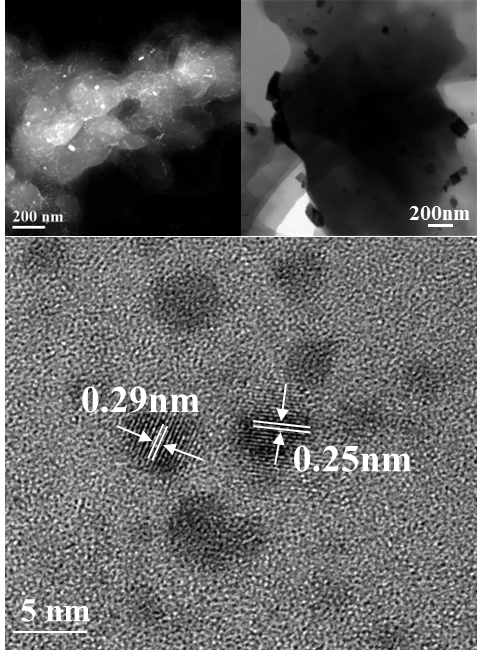
**

**Figure S11.** STEM and HRTEM images of LiPb-OA/OLAM.


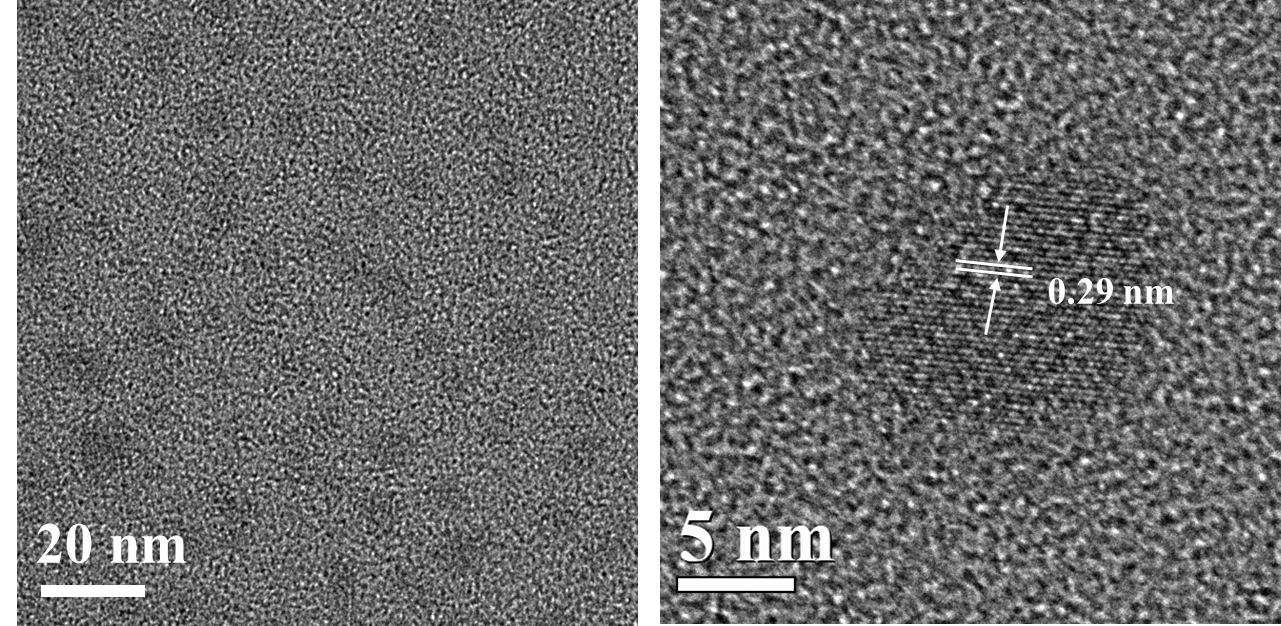


**Figure S12.** HRTEM images of Li_17_Pb_4_ NCs.

**
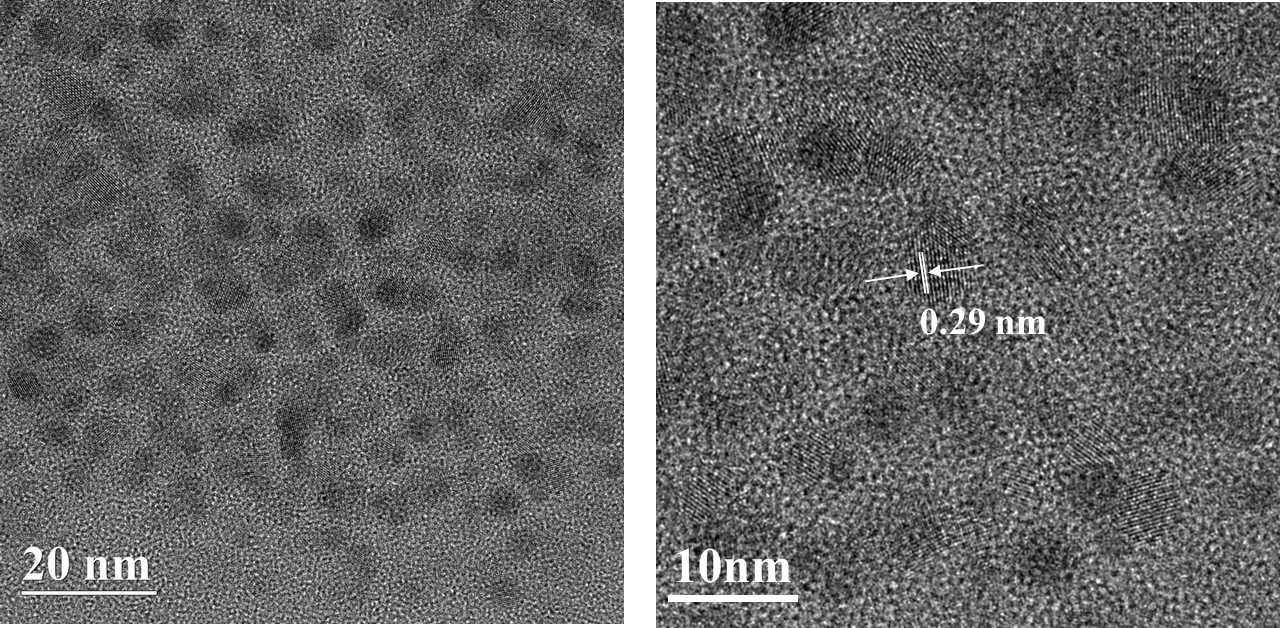
**

**Figure S13.** HRTEM images of CsPbBr_3_:9Li⁺ NCs.

**
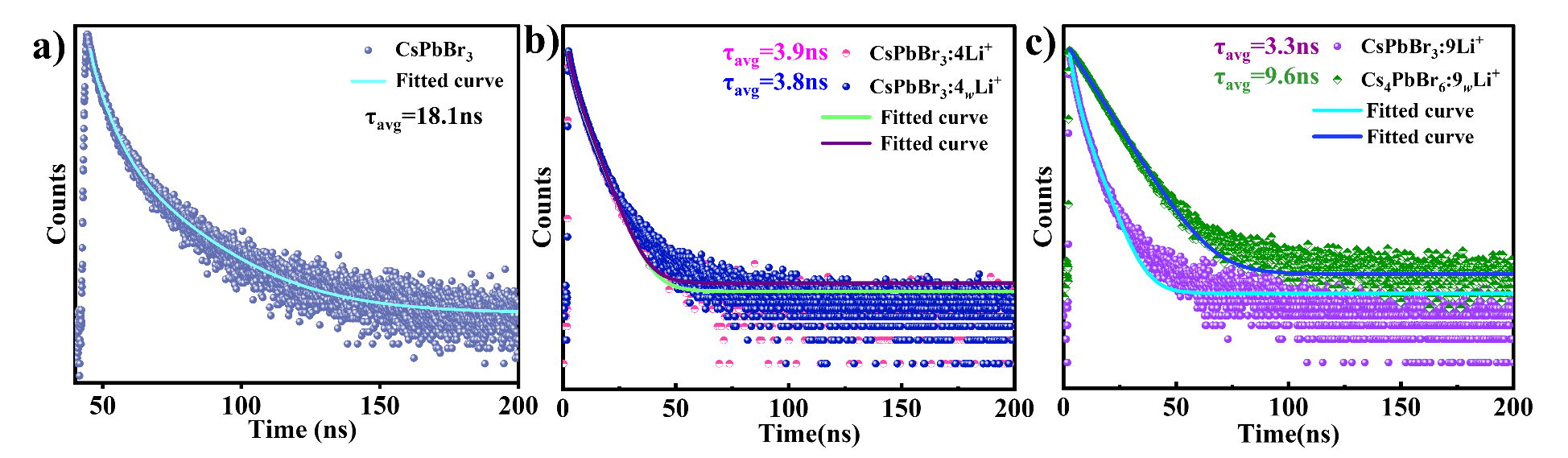
**

**Figure S14.** PL Lifetime characterizations of pure CsPbBr_3_ NCs (a), CsPbBr_3_:4Li^+^ NCs, CsPbBr_3_:4*_w_*Li^+^ NCs (b) and CsPbBr_3_:9Li^+^ NCs, Cs_4_PbBr_6_:9*_w_*Li^+^ NCs (c).

**
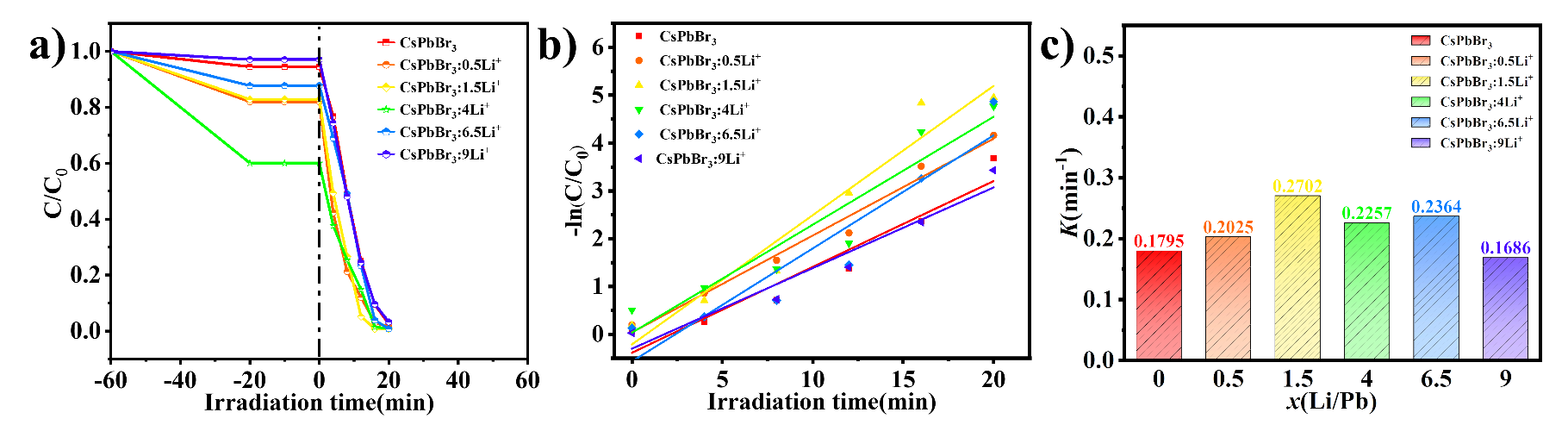
**

**Figure S15.** Photodegradation efficiency of pure and various CsPbBr_3_:Li^+^ NCs are presented , where the RhB concentration changes with irradiation time (a) and their kinetic curves are presented in (b) and (c). The rate of degradation is presented in the below Table S6. The highest degradation rate was observed for CsPbBr_3_:1.5Li^+^ NCs.

**Table S7.** Rate of degradation of the of pure and various CsPbBr_3_:Li^+^ NCs.

| Type of NCs | k (min^-1^) |
| --- | --- |
| CsPbBr_3_ NCs | 0.1795 |
| CsPbBr_3_:0.5Li^+^ NCs | 0.2025 |
| CsPbBr_3_:1.5Li^+^ NCs | 0.2702 |
| CsPbBr_3_:4Li^+^ NCs | 0.2257 |
| CsPbBr_3_:6.5Li^+^ NCs | 0.2364 |
| CsPbBr_3_:9Li^+^ NCs | 0.1686 |


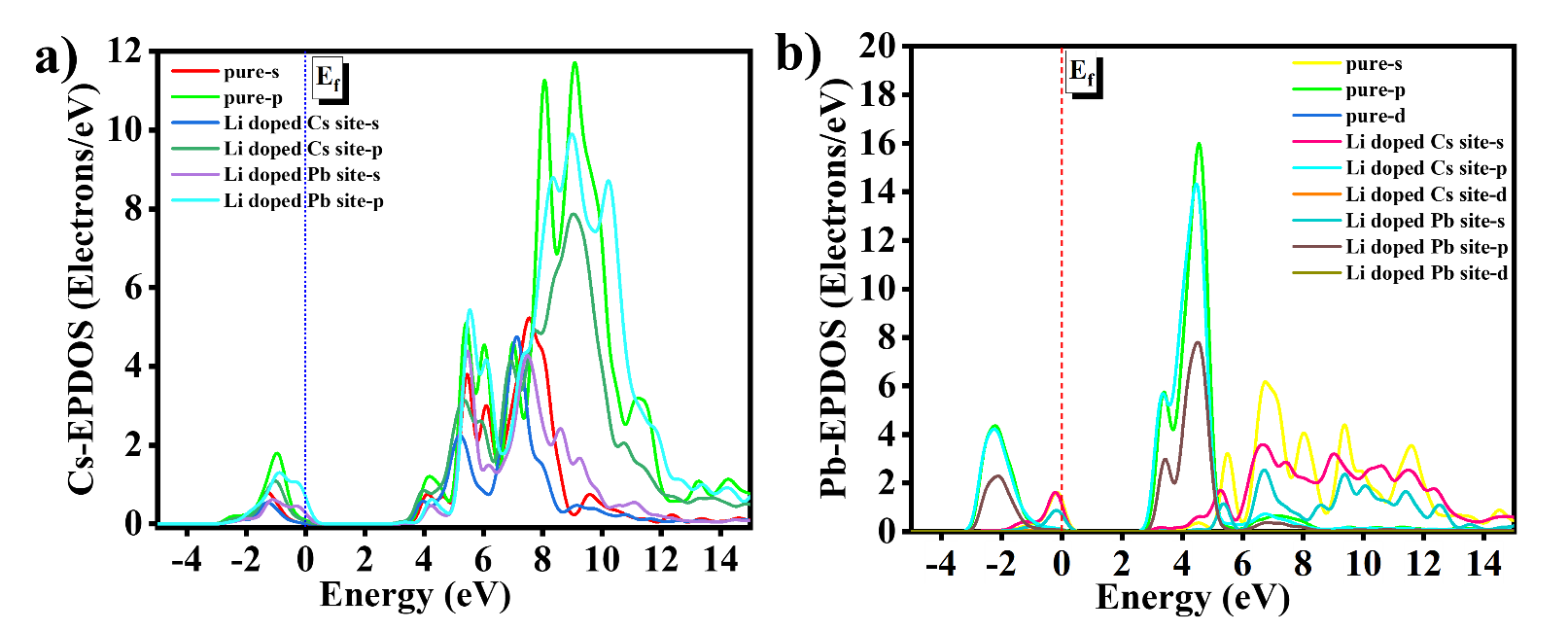


**Figure S16.** Total and EPDoS of pure and doped systems: (a) Cs-EPDoS, (b) Pb-EPDoS.

**Figure S17.** The CIE distribution diagram of CsPbBr_3_:9Li^+^ NCs green LEDs. As the current increases, the color coordinates change from (0.0557,0.7113) to (0.0684,0.7105).

**Table S8.** The luminous efficacy of CsPbBr_3_ NCs CsPbBr_3_:0.5Li^+^ NCs, CsPbBr_3_:4Li^+^ NCs, CsPbBr_3_:6.5Li^+^ NCs and CsPbBr_3_:9Li^+^ NCs under different currents.

| Luminous efficacy (lm/W)  Current(mA) | CsPbBr_3_ NCs | CsPbBr_3_:0.5Li^+^ NCs | CsPbBr_3_:4Li^+^ NCs | CsPbBr_3_:6.5Li^+^ NCs | CsPbBr_3_:9Li^+^ NCs |
| --- | --- | --- | --- | --- | --- |
| 20 | 96.55 | 88.53 | 112.48 | 106.6 | 110.08 |
| 40 | 91.07 | 88.37 | 106.72 | 106.36 | 102.32 |
| 60 | 91.64 | 87.29 | 103.71 | 106.25 | 99.91 |
| 80 | 86.54 | 83.43 | 98.79 | 103.61 | 95.35 |
| 100 | 86.77 | 78.09 | 101.7 | 102.3 | 98.78 |
| 120 | 81.73 | 74.91 | 98.8 | 99.97 | 95.98 |
| 160 | 81.11 | 70.88 | 94.87 | 94.95 | 93.15 |
| 200 | 77.56 | 68.84 | 91.5 | 91.54 | 87.59 |
| 240 | 76.23 | 64.49 | 88.47 | 84.99 | 83.64 |
| 280 | 73.23 | 55.44 | 76.09 | 69.21 | 83.94 |
| 320 | 71.51 | 46.26 | 67.28 | 61.48 | 82.33 |

**Table S9.** Comparison of luminous efficacy reported in the literature with our work.

| **Luminescent materials** | **Luminous efficacy[lm W^-1^]** | **References** |
| --- | --- | --- |
| LDH/CsPbBr_3_ | 58.43 | [7] |
| B-/G-/R-CQDs | 31.3 | [8] |
| CsPb_1-x_Sn_x_Br_3_ | 37.70 | [9] |
| CIS/ZnS | 32.7 | [10] |
| CsPbX_3_ NCs | 46 | [11] |
| Mn:Cs_2_ZnX_4_(X=Cl,Br,I) | 32 | [12] |
| Ba_0.93_Eu_0.07_Al_2_O_4_ | 20 | [13] |
| Mn-doped C_4_H_12_N_2_ZnX_4_ | 98.52 | [14] |
| CsPbBr_3_ NCs-MS | 47.6 | [15] |
| InGaN | 41 | [16] |
| CsPbBr_3_-SiO_2_ | 38.80 | [17] |
| BDGA-CsPbBr_3_ | 93.5 | [18] |
| Cs_2_Ag_0.6_Na_0.4_InCl_6_ | 20.1 | [19] |
| CsPbBr_3_ PQD/polymer | 46.62 | [20] |
| MAPbX_3_ | 109 | [21] |
| CsPbBr_3_/PMA | 58.4 | [22] |
| CsPbBr_3_ NCs | 56.6 | [23] |
| CsPbBr_3_@SiO_2_@PS | 96 | [24] |
| (C_9_NH_20_)_9_[Pb_3_Br_11_](MnBr_4_)_2_ | 80.93 | [25] |

**Table S10.** CsPbBr_3_ NCs, CsPbBr_3_:0.5Li^+^ NCs, CsPbBr_3_:4Li^+^ NCs, CsPbBr_3_:6.5Li^+^ NCs and CsPbBr_3_:9Li^+^ NCs based WLED device, and their Coordinates corresponding to the 1931 Gamut (NTCS) International Commission on Illumination (CIE).

| Type of NCs | 1931 Gamut (NTSC %) |
| --- | --- |
| CsPbBr_3_ NCs | 110.2 |
| CsPbBr_3_:0.5Li^+^ NCs | 113.8 |
| CsPbBr_3_:4Li^+^ NCs | 109.1 |
| CsPbBr_3_:6.5Li^+^ NCs | 119.4 |
| CsPbBr_3_:9Li^+^ NCs | 114.2 |

**Table S11.** CsPbBr_3_ NCs, CsPbBr_3_:0.5Li^+^ NCs, CsPbBr_3_:4Li^+^ NCs, CsPbBr_3_:6.5Li^+^ NCs and CsPbBr_3_:9Li^+^ NCs based WLED device, and their Coordinates corresponding to the 1931 Gamut (Rec.2020 %) international Commission on Illumination (CIE).

| Type of NCs | 1931 Gamut (Rec.2020 %) |
| --- | --- |
| CsPbBr_3_ NCs | 82.3% |
| CsPbBr_3_:0.5Li^+^ NCs | 85.0% |
| CsPbBr_3_:4Li^+^ NCs | 81.4% |
| CsPbBr_3_:6.5Li^+^ NCs | 89.1% |
| CsPbBr_3_:9Li^+^ NCs | 85.2% |

**Table S12.** The color rendering index (Ra) of CsPbBr_3_ NCs under different currents as well as R1(Light Red), R2(Gray - Yellow), R3(Saturated Yellow), R4(Light Green), R5(Saturated Green), R6(Light Blue), R7(Saturated Blue), R8(Light Purple), R9(Saturated Red), R10(Saturated Yellow - Green), R11(Saturated Green - Blue), R12(Saturated Blue - Purple), R13(White - skinned C - Tone), R14(Leaf Green), R15(Asian Skin Tone). These Ra to R15 representations are same for the next samples.

| Current(mA) | Ra | R1 | R2 | R3 | R4 | R5 | R6 | R7 | R8 | R9 | R10 | R11 | R12 | R13 | R14 | R15 |
| --- | --- | --- | --- | --- | --- | --- | --- | --- | --- | --- | --- | --- | --- | --- | --- | --- |
| 20 | 90.7 | 94 | 91 | 89 | 89 | 94 | 91 | 88 | 90 | 85 | 79 | 94 | 76 | 92 | 93 | 91 |
| 40 | 91.7 | 97 | 94 | 92 | 91 | 93 | 90 | 88 | 90 | 89 | 84 | 94 | 76 | 96 | 95 | 93 |
| 60 | 92 | 98 | 95 | 92 | 90 | 93 | 90 | 88 | 90 | 94 | 85 | 94 | 76 | 96 | 95 | 93 |
| 80 | 92 | 98 | 95 | 92 | 90 | 93 | 89 | 88 | 91 | 93 | 86 | 94 | 76 | 96 | 95 | 93 |
| 100 | 93 | 96 | 94 | 92 | 91 | 95 | 93 | 90 | 92 | 92 | 85 | 96 | 76 | 95 | 95 | 93 |
| 120 | 92.1 | 98 | 96 | 93 | 91 | 93 | 89 | 87 | 90 | 97 | 87 | 94 | 77 | 97 | 96 | 93 |
| 160 | 92.3 | 98 | 96 | 94 | 91 | 93 | 89 | 88 | 90 | 97 | 88 | 94 | 77 | 96 | 96 | 93 |
| 200 | 94 | 98 | 95 | 92 | 92 | 96 | 95 | 91 | 92 | 98 | 88 | 97 | 77 | 96 | 95 | 94 |
| 240 | 92.7 | 98 | 96 | 92 | 91 | 95 | 92 | 88 | 90 | 95 | 88 | 95 | 77 | 96 | 95 | 92 |
| 280 | 93.5 | 98 | 95 | 91 | 91 | 96 | 95 | 90 | 91 | 97 | 86 | 96 | 78 | 96 | 94 | 93 |
| 320 | 93.4 | 98 | 95 | 91 | 91 | 97 | 95 | 90 | 91 | 96 | 86 | 96 | 78 | 96 | 94 | 93 |

**Table S13.** The color rendering index (Ra) of CsPbBr_3_:0.5Li^+^ NCs under different currents.

| Current(mA) | Ra | R1 | R2 | R3 | R4 | R5 | R6 | R7 | R8 | R9 | R10 | R11 | R12 | R13 | R14 | R15 |
| --- | --- | --- | --- | --- | --- | --- | --- | --- | --- | --- | --- | --- | --- | --- | --- | --- |
| 20 | 92.3 | 91 | 93 | 95 | 93 | 92 | 91 | 93 | 90 | 74 | 82 | 88 | 78 | 91 | 96 | 90 |
| 40 | 94 | 92 | 94 | 96 | 95 | 94 | 93 | 96 | 92 | 78 | 86 | 90 | 77 | 92 | 97 | 91 |
| 60 | 95.1 | 93 | 94 | 97 | 96 | 95 | 94 | 98 | 93 | 81 | 88 | 92 | 77 | 92 | 98 | 91 |
| 80 | 96 | 93 | 95 | 98 | 98 | 96 | 94 | 99 | 94 | 86 | 91 | 94 | 75 | 93 | 98 | 92 |
| 100 | 95.8 | 93 | 95 | 98 | 99 | 96 | 94 | 98 | 94 | 87 | 92 | 96 | 75 | 92 | 99 | 91 |
| 120 | 97.1 | 96 | 98 | 99 | 97 | 97 | 96 | 98 | 96 | 90 | 95 | 92 | 77 | 96 | 99 | 95 |
| 160 | 94.6 | 94 | 95 | 98 | 96 | 94 | 90 | 95 | 95 | 94 | 95 | 97 | 71 | 93 | 100 | 92 |
| 200 | 97.7 | 98 | 99 | 96 | 97 | 99 | 95 | 99 | 99 | 98 | 97 | 94 | 76 | 99 | 98 | 98 |
| 240 | 93.5 | 95 | 97 | 97 | 92 | 90 | 87 | 94 | 95 | 99 | 97 | 94 | 63 | 94 | 99 | 92 |
| 280 | 96.8 | 98 | 97 | 95 | 98 | 96 | 94 | 99 | 98 | 95 | 93 | 96 | 69 | 98 | 97 | 97 |
| 320 | 96 | 97 | 96 | 94 | 97 | 95 | 93 | 99 | 97 | 91 | 90 | 95 | 68 | 97 | 96 | 96 |

**Table S14.** The color rendering index (Ra) of CsPbBr_3_:4Li^+^ NCs under different currents.

| Current(mA) | Ra | R1 | R2 | R3 | R4 | R5 | R6 | R7 | R8 | R9 | R10 | R11 | R12 | R13 | R14 | R15 |
| --- | --- | --- | --- | --- | --- | --- | --- | --- | --- | --- | --- | --- | --- | --- | --- | --- |
| 20 | 84.7 | 85 | 91 | 92 | 79 | 79 | 79 | 91 | 82 | 44 | 69 | 69 | 78 | 87 | 94 | 85 |
| 40 | 85.8 | 83 | 89 | 91 | 83 | 84 | 84 | 92 | 80 | 46 | 69 | 73 | 80 | 84 | 94 | 82 |
| 60 | 87 | 84 | 90 | 93 | 84 | 85 | 86 | 93 | 81 | 49 | 72 | 74 | 82 | 85 | 95 | 83 |
| 80 | 87.6 | 79 | 85 | 91 | 93 | 93 | 94 | 89 | 77 | 51 | 70 | 85 | 79 | 77 | 94 | 77 |
| 100 | 89.4 | 86 | 92 | 97 | 85 | 87 | 89 | 95 | 84 | 56 | 79 | 77 | 83 | 87 | 97 | 85 |
| 120 | 87.8 | 78 | 85 | 92 | 97 | 95 | 92 | 87 | 76 | 54 | 73 | 88 | 79 | 76 | 95 | 76 |
| 160 | 87.2 | 78 | 85 | 94 | 98 | 94 | 88 | 85 | 75 | 58 | 76 | 92 | 76 | 76 | 96 | 75 |
| 200 | 92.4 | 90 | 96 | 94 | 90 | 92 | 93 | 97 | 87 | 66 | 93 | 85 | 76 | 91 | 96 | 87 |
| 240 | 92.1 | 90 | 96 | 93 | 90 | 92 | 93 | 97 | 87 | 66 | 94 | 85 | 76 | 91 | 95 | 86 |
| 280 | 88.4 | 85 | 92 | 91 | 85 | 87 | 90 | 95 | 82 | 52 | 88 | 80 | 81 | 86 | 94 | 79 |
| 320 | 84.6 | 80 | 89 | 88 | 81 | 83 | 86 | 94 | 76 | 33 | 84 | 77 | 78 | 81 | 91 | 71 |

**Table S15.** The color rendering index (Ra) of CsPbBr_3_:6.5Li^+^ NCs under different currents.

| Current(mA) | Ra | R1 | R2 | R3 | R4 | R5 | R6 | R7 | R8 | R9 | R10 | R11 | R12 | R13 | R14 | R15 |
| --- | --- | --- | --- | --- | --- | --- | --- | --- | --- | --- | --- | --- | --- | --- | --- | --- |
| 20 | 87.4 | 89 | 88 | 88 | 87 | 87 | 84 | 89 | 87 | 64 | 70 | 87 | 76 | 87 | 93 | 87 |
| 40 | 89 | 89 | 89 | 90 | 90 | 89 | 87 | 90 | 87 | 67 | 74 | 89 | 78 | 88 | 94 | 88 |
| 60 | 90 | 90 | 90 | 91 | 91 | 90 | 88 | 91 | 89 | 70 | 76 | 89 | 79 | 89 | 95 | 89 |
| 80 | 91 | 92 | 92 | 92 | 91 | 90 | 88 | 92 | 91 | 74 | 79 | 89 | 80 | 91 | 95 | 91 |
| 100 | 91 | 91 | 91 | 92 | 92 | 91 | 89 | 92 | 90 | 74 | 79 | 90 | 81 | 90 | 95 | 90 |
| 120 | 91.9 | 91 | 91 | 92 | 93 | 93 | 92 | 93 | 90 | 75 | 80 | 91 | 82 | 90 | 95 | 90 |
| 160 | 92.8 | 90 | 91 | 92 | 95 | 96 | 95 | 94 | 90 | 78 | 81 | 94 | 82 | 89 | 95 | 89 |
| 200 | 92.4 | 88 | 89 | 91 | 96 | 97 | 98 | 93 | 88 | 77 | 80 | 97 | 82 | 86 | 94 | 87 |
| 240 | 88.8 | 84 | 86 | 88 | 92 | 93 | 93 | 90 | 84 | 77 | 76 | 98 | 80 | 81 | 92 | 83 |
| 280 | 95.6 | 94 | 96 | 99 | 96 | 95 | 95 | 97 | 93 | 81 | 93 | 94 | 75 | 94 | 99 | 92 |
| 320 | 94.6 | 92 | 95 | 98 | 94 | 93 | 93 | 98 | 92 | 77 | 90 | 90 | 82 | 93 | 99 | 90 |

**Table S16.** The color rendering index (Ra) of CsPbBr_3_:9Li^+^ NCs under different currents.

| Current(mA) | Ra | R1 | R2 | R3 | R4 | R5 | R6 | R7 | R8 | R9 | R10 | R11 | R12 | R13 | R14 | R15 |
| --- | --- | --- | --- | --- | --- | --- | --- | --- | --- | --- | --- | --- | --- | --- | --- | --- |
| 20 | 84.1 | 86 | 87 | 89 | 85 | 80 | 76 | 86 | 84 | 52 | 66 | 82 | 70 | 86 | 94 | 84 |
| 40 | 86 | 86 | 87 | 89 | 86 | 83 | 80 | 90 | 86 | 55 | 68 | 85 | 73 | 85 | 94 | 84 |
| 60 | 87 | 87 | 87 | 90 | 87 | 84 | 82 | 91 | 87 | 58 | 70 | 85 | 74 | 86 | 94 | 85 |
| 80 | 87.7 | 88 | 88 | 90 | 88 | 86 | 83 | 92 | 88 | 61 | 71 | 86 | 75 | 87 | 94 | 85 |
| 100 | 87.3 | 88 | 88 | 89 | 86 | 84 | 82 | 92 | 89 | 60 | 70 | 85 | 75 | 87 | 94 | 86 |
| 120 | 87.5 | 85 | 86 | 88 | 89 | 87 | 86 | 93 | 86 | 58 | 68 | 87 | 77 | 84 | 93 | 83 |
| 160 | 88.3 | 87 | 88 | 91 | 90 | 87 | 85 | 92 | 87 | 61 | 72 | 87 | 79 | 86 | 95 | 85 |
| 200 | 89.4 | 85 | 86 | 90 | 91 | 89 | 89 | 98 | 87 | 62 | 72 | 90 | 78 | 83 | 94 | 82 |
| 240 | 89.2 | 87 | 89 | 93 | 90 | 88 | 86 | 94 | 87 | 61 | 75 | 87 | 80 | 87 | 96 | 85 |
| 280 | 89 | 84 | 89 | 98 | 89 | 86 | 87 | 96 | 84 | 51 | 78 | 85 | 78 | 85 | 98 | 80 |
| 320 | 87.4 | 82 | 87 | 98 | 87 | 84 | 85 | 96 | 80 | 41 | 76 | 83 | 77 | 82 | 98 | 76 |

**References**

[1] R. Begum, M. R. Parida, A. L. Abdelhady, B. Murali, N. M. Alyami, G. H. Ahmed, M. N. Hedhili, O. M. Bakr, O. F. Mohammed, *Journal of the American Chemical Society* **2017**, *139* (2), 731.

[2] Z. He, X. Liang, W. Xiang, *Chemical Engineering Journal* **2022**, *427*, 130964.

[3] D. Amgar, T. Binyamin, V. Uvarov, L. Etgar, *Nanoscale* **2018**, *10* (13), 6060.

[4] M. Li, J. Xu, Y. Song, F. Chen, *CrystEngComm* **2022**, *24* (45), 7962.

[5] W. van der Stam, J. J. Geuchies, T. Altantzis, K. H. W. van den Bos, J. D. Meeldijk, S. Van Aert, S. Bals, D. Vanmaekelbergh, C. de Mello Donega, *Journal of the American Chemical Society* **2017**, *139* (11), 4087.

[6] S. Shyamal, S. K. Dutta, N. Pradhan, *The Journal of Physical Chemistry Letters* **2019**, *10* (24), 7965.

[7] Y. Liu, B. Shi, Q. Liu, C. Lü, *Journal of Alloys and Compounds* **2020**, *843*, 155819.

[8] Z. Wang, F. Yuan, X. Li, Y. Li, H. Zhong, L. Fan, S. Yang, *Advanced Materials* **2017**, *29* (37), 1702910.

[9] S. Liu, G. Shao, L. Ding, J. Liu, W. Xiang, X. Liang, *Chemical Engineering Journal* **2019**, *361*, 937.

[10] P.-H. Chuang, C. C. Lin, R.-S. Liu, *ACS Applied Materials & Interfaces* **2014**, *6* (17), 15379.

[11] H. Xu, J. Wang, T. Xuan, C. Lv, J. Hou, L. Zhang, Y. Dong, J. Shi, *Chemical Engineering Journal* **2019**, *364*, 20.

[12] K. Li, Y. Ye, W. Zhang, Y. Zhou, Y. Zhang, S. Lin, H. Lin, J. Ruan, C. Liu, *Nano Research* **2022**, *15* (10), 9368.

[13] X. Li, J. D. Budai, F. Liu, J. Y. Howe, J. Zhang, X.-J. Wang, Z. Gu, C. Sun, R. S. Meltzer, Z. Pan, *Light: Science & Applications* **2013**, *2* (1), e50.

[14] Y.-P. Lin, S. Hu, J. Xu, Z. Zhang, X. Qi, X. Lu, J. Jin, X.-Y. Huang, Q. Xu, Z. Deng, Z. Xiao, K.-Z. Du, *Chemical Engineering Journal* **2023**, *468*, 143818.

[15] X. Di, L. Shen, J. Jiang, M. He, Y. Cheng, L. Zhou, X. Liang, W. Xiang, *Journal of Alloys and Compounds* **2017**, *729*, 526.

[16] E. Jang, S. Jun, H. Jang, J. Lim, B. Kim, Y. Kim, *Advanced Materials* **2010**, *22* (28), 3076.

[17] S. Yoon, M. Seo, I. S. Kim, K. Lee, K. Woo, *Small* **2023**, *19* (7), 2206311.

[18] Y. Zhang, G. Li, G. Hou, J. Lin, M. Chen, S. Liu, H. Lin, J. Fang, C. Jing, J. Chu, *Chemical Engineering Journal* **2022**, *438*, 135270.

[19] H. Peng, W. Huang, L. Kong, S. Yu, B. Zou, *Advanced Functional Materials* **2025**, *n/a* (n/a), 2422745.

[20] Y. Li, A. Alam, T. Zhou, C. Wang, Y. Wang, T. Li, *Advanced Functional Materials* **2025**, *35* (4), 2413963.

[21] Q. Zhou, Z. Bai, W.-g. Lu, Y. Wang, B. Zou, H. Zhong, *Advanced Materials* **2016**, *28* (41), 9163.

[22] P. R. Bommireddy, J. B. B, S. Sunku, K. B. C, Y. Suh, C. S. M, S.-H. Park, *Heliyon* **2024**, *10* (2).

[23] H. Wu, S. Wang, F. Cao, J. Zhou, Q. Wu, H. Wang, X. Li, L. Yin, X. Yang, *Chemistry of Materials* **2019**, *31* (6), 1936.

[24] S. Wang, D. Chen, K. Xu, J. Hu, D. Huang, M. Hong, H. Zhu, *Nano Research* **2023**, *16* (7), 10507.

[25] M. Li, J. Zhou, G. Zhou, M. S. Molokeev, J. Zhao, V. Morad, M. V. Kovalenko, Z. Xia, *Angewandte Chemie International Edition* **2019**, *58* (51), 18670.
